# Interfacial Engineering at Quantum Dot-Sensitized TiO_2_ Photoelectrodes for Ultrahigh Photocurrent Generation

**DOI:** 10.1021/acsami.0c19352

**Published:** 2021-02-01

**Authors:** Tea-Yon Kim, Byung Su Kim, Jong Gyu Oh, Seul Chan Park, Jaeyoung Jang, Thomas W. Hamann, Young Soo Kang, Jin Ho Bang, Sixto Giménez, Yong Soo Kang

**Affiliations:** † Department of Chemistry, Michigan State University, East Lansing, Michigan 48824-1322, United States; ‡ Department of Energy Engineering and Center for Next Generation Dye-Sensitized Solar Cells, Hanyang University, Seoul 04763, Korea; § Department of Energy Engineering, Hanyang University, Seoul 04763, Korea; ∥ Korea Center for Artificial Photosynthesis and Department of Chemistry, Sogang University, Seoul 04107, Korea; ⊥ Department of Chemical and Molecular Engineering and Department of Applied Chemistry, Center for Bionano Intelligence Education and Research, Hanyang University, Ansan 15588, Gyeonggi-do, Korea; # Institute of Advanced Materials (INAM), Universitat Jaume I, Castelló 12006, Spain

**Keywords:** TiO_2_/QD, photoanode, photoelectrochemical cells, surface passivation layer, surface state, charge collection, photocurrent density

## Abstract

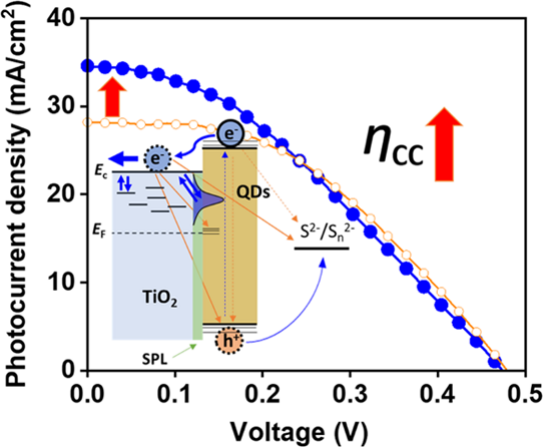

Metal oxide semiconductor/chalcogenide quantum dot (QD) heterostructured photoanodes show photocurrent densities >30 mA/cm^2^ with ZnO, approaching the theoretical limits in photovoltaic (PV) cells. However, comparative performance has not been achieved with TiO_2_. Here, we applied a TiO_2_(B) surface passivation layer (SPL) on TiO_2_/QD (PbS and CdS) and achieved a photocurrent density of 34.59 mA/cm^2^ under AM 1.5G illumination for PV cells, the highest recorded to date. The SPL improves electron conductivity by increasing the density of surface states, facilitating multiple trapping/detrapping transport, and increasing the coordination number of TiO_2_ nanoparticles. This, along with impeded electron recombination, led to enhanced collection efficiency, which is a major factor for performance. Furthermore, SPL-treated TiO_2_/QD photoanodes were successfully exploited in photoelectrochemical water splitting cells, showing an excellent photocurrent density of 14.43 mA/cm^2^ at 0.82 V versus the Reversible Hydrogen Electrode (RHE). These results suggest a new promising strategy for the development of high-performance photoelectrochemical devices.

## 1 Introduction

Chalcogenide quantum dots (QDs) have attracted much attention as building blocks for next-generation light-harvesting devices due to their outstanding optical characteristics such as a wide light absorption range over the near-IR regions and high extinction coefficient.^1−5^ Typical examples of such light-harvesting devices include photovoltaic (PV) cells and photoelectrochemical (PEC) water splitting cells, consisting of light-harvesting materials deposited on a mesoporous n-type semiconductor layer of TiO_2_, ZnO, or SnO_2_; an electrolyte; and a counter electrode.^6−8^

**Figure 1:**
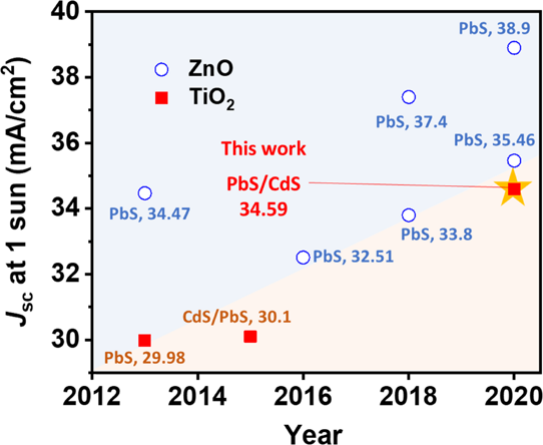
Short-circuit current density (*J*_sc_) for PV cells since 2013 with semiconductor/chalcogenide QD photoanodes with ZnO (blue empty circles) and TiO_2_ (red filled squares) at a 1 sun condition.^9,11−17^ The yellow star indicates the performance of this work with TiO_2_.

Over the last 5 years, high photocurrent densities greater than 30 mA/cm^2^ have been reported for PV cells using metal oxide/chalcogenide QD heterostructured photoanodes having high light-harvesting ability (Figure 1). For instance, ZnO/chalcogenide QDs have led to ultrahigh photocurrent densities in PV cells, with a record value of 39 mA/cm^2^, closely approaching the theoretical photocurrent density (44 mA/cm^2^) for the 1.1 eV band gap of such QDs.^9^ However, TiO_2_/chalcogenide QD heterostructures have been struggling to reach photocurrent densities greater than 30 mA/cm^2^, mostly due to the lower electron conductivity of TiO_2_ compared to ZnO.^10^ In this work, we achieved a photocurrent density close to 35 mA/cm^2^, the highest recorded to date (Figure 1).

Chalcogenide QDs have been actively exploited for the generation of solar hydrogen in PEC water splitting cells due to their outstanding water durability compared to other light-harvesting materials such as metalorganic dyes and halide perovskites.^18,19^ In this regard, TiO_2_/chalcogenide QD photoanodes are much more attractive for PEC hydrogen generation compared to ZnO/chalcogenide QDs due to the excellent stability of TiO_2_ in strong-base electrolyte conditions.^20^ Therefore, the development of TiO_2_/chalcogenide QD photoanodes showing a high photocurrent density greater than 30 mA/cm^2^ in PV cells is extremely attractive for PEC applications.

To design the optimal architecture of photoanodes with TiO_2_/chalcogenide QDs for high photocurrents, enhancement of both charge transfer kinetics at the TiO_2_/chalcogenide QD interface and charge transport via the TiO_2_ films should be simultaneously considered.^21^ As a general strategy, the introduction of a surface passivation layer (SPL) on TiO_2_ film has led to significant improvements in sensitized PV cells.^22,23^ Particularly, TiCl_4_ treatment to form a TiO_2_ SPL at the interface of TiO_2_/light-harvesting materials has led to an about 10–30% photocurrent density increase.^24^ In dye-sensitized solar cells (DSSCs) with a SPL, performance enhancement has been attributed to the improved electron injection, mostly because of driving force enhancement through the conduction band (CB) shift of TiO_2_ and suppression of electron recombination at the TiO_2_/dye interface.^24^ However, changes in electron transport induced by SPLs have been reported to be minimal due to the high diffusion length of electrons through the mesoporous TiO_2_ layer (20–25 μm with I_3_^–^/I^–^ redox couples and 10–12.5 μm for water oxidation^25,26^) compared to the film thickness (∼13 μm).^24,27,28^ Conversely, multiple electron recombination pathways have been reported for TiO_2_/chalcogenide QD photoanodes due to the complex distribution of energy states as well as the ultrafast electron injection rate to TiO_2_ (<10^–9^ s).^3,18,29^ Therefore, it is expected that the effects of the SPL on the functional performance of TiO_2_/QD photoanodes will be significantly different from those of conventional TiO_2_/dye photoanodes, although explicit effects on transport properties have not been completely elucidated.

Electron transport in mesoporous TiO_2_ films is commonly interpreted with the trap state-mediated transport model with multiple trapping/detrapping events.^30^ In semiconductors, shallow traps in a band gap significantly affect electron transport and are typically located 0.5–1.0 eV below the CB.^31^ Therefore, the deposition of the SPL impacts the population and distribution of trap states of TiO_2_ with a concomitant effect on the TiO_2_/chalcogenide QD interface. During SPL formation on the TiO_2_ film, the surface states, which are localized and generated by chemical surface treatment, can affect electron transport.^32^ The surface states of TiO_2_ are formed mainly at the trap state area 0.3–0.4 eV below the CB and originate mainly from undercoordinated Ti^4+^ atoms on the anatase TiO_2_ surface.^33^ It is well-known that charge transfer at the interface of the mesoporous semiconductor/solution is affected by surface states.^34−36^ However, even though the increase in the density of surface trap states in relation to deposition of the TiO_2_ SPL has been extensively reported, its effects on electron transport through mesoporous TiO_2_ films are uncertain.^24,28,37^ Additionally, changes in film morphology during the formation of the SPL have a significant impact on the electron transport of TiO_2_ films.^30^ Therefore, the effects of the SPL on electron transport, as well as on energetics and the morphology of the photoelectrodes, should be precisely tuned to improve the photocurrent density.

Herein, we achieved a photocurrent density of 34.59 mA/cm^2^ in PV cells with a 0.18 cm^2^ active area and 14.43 mA/cm^2^ in PEC water splitting cells with an active area of 1.33 cm^2^ at 1 sun condition with about 20% enhancement (compared to the reference samples) by hydrothermal treatment of TiCl_4_ to form an SPL on the TiO_2_/PbS-CdS QD photoanodes. Based on the results of photocurrent densities on PV cells and PEC water splitting cells, the SPL facilitates electron conductivity of mesoporous TiO_2_ films by increasing the density of surface states and the coordination number of TiO_2_ nanoparticles (NPs). The increased electron conductivity is mostly due to an increase in surface states for multiple trapping/detrapping transport through TiO_2_ films, which was quantified in terms of the chemical diffusion coefficient. The suppression of electron recombination was also observed for the SPL. Therefore, we conclude that the main effects of the SPL on the photocurrent density of TiO_2_/PbS-CdS QD photoanodes are related to improved charge collection efficiency (η_cc_), driven by enhancement of electron transport and suppression of electron recombination.

## 2 Experimental Section

### 2.1 Materials

All chemicals and materials were purchased from a commercial company and were used without further purification. Acetonitrile (ACN), acetone (ACT), deionized water (DI water), ethanol (EtOH), methanol (MeOH), cadmium acetate dihydrate (Cd(CH_3_COO)_2_·2H_2_O), copper(II) sulfate (CuSO_4_), hydrochloride acid (HCl), lead nitrate (Pb(NO_3_)_2_), potassium chloride (KCl), sulfur (S), sodium sulfide (Na_2_S), sodium sulfide nonahydrate (Na_2_S·9H_2_O), sodium sulfite (Na_2_SO_3_), sodium thiosulfate (Na_2_S_2_O_3_), titanium(IV) chloride (TiCl_4_), and zinc acetate dihydrate (Zn(CH_3_COO)_2_·2H_2_O) were purchased from Sigma Aldrich. Fluorine-doped tin oxide (FTO) glass substrates (TEC-15) were purchased from Pilkington (U.K.). Titanium dioxide (TiO_2_) nanoparticulate pastes were purchased from Dyesol (30NR-T, Australia) and CCIC (PST-18 NR, PST-400C, Japan).

### 2.2 Preparation of Mesoporous TiO_2_/PbS-CdS QD Photoanodes and Counter Electrodes

An FTO glass substrate was cleaned in a two-step sonication process with an aqueous detergent solution for 1 h (2% Hellmanex III in DI water, v/v) and subsequently with an organic solvent mixture (EtOH and ACT, 1:1, v/v). The cleaned FTO substrates were used for all working- and counter electrodes. For the purification of TiCl_4_, a 0.05 mol of TiCl_4_ solution was slowly added dropwise to an HCl solution in a 1:5 ratio at −20 °C.^38^ A TiO_2_ blocking layer (∼20 nm) on the FTO substrate of the working electrode was treated using the hydrothermal method with a 40 mM TiCl_4_ aqueous solution at 70 °C for 30 min. After rinsing with DI water and EtOH, the working electrode was sintered at 450 °C for 30 min. This process was repeated to form double blocking layers. Next, two layers of TiO_2_ NPs and one layer of TiO_2_ light-scattering particles (∼400 nm, CCIC) were doctor-bladed onto the blocking layer-coated FTO glass and sintered at 450 °C for 30 min. The thickness of the photoanode was around 12 μm. For the PT20 and PT30 samples, TiCl_4_ treatment was conducted on neat TiO_2_ films with 20 and 30 nm NPs (termed T20 and T30, hereafter) using the hydrothermal method with a 40 mM TiCl_4_ aqueous solution at 70 °C for 18 min and then sintered at 450 °C for 30 min before rinsing with DI water and EtOH.

For the photoanode used in both PV cells and PEC water splitting cells, the co-sensitization of PbS and CdS QDs was applied in this work using the successive ionic layer adsorption and reaction (SILAR) method for the direct growth of QDs on the surface of TiO_2_ films. The detailed synthetic method of QDs was followed according to our previous study with slight modifications.^39^ First, the TiO_2_ films were immersed in a 0.1 M Cd(CH_3_COO)_2_·2H_2_O solution in MeOH for a few seconds for the precoating of QDs. For PbS QD deposition, the films were immersed in a Pb^2+^ (0.02 M Pb(NO_3_)_2_ in MeOH) and a S^2–^ solution (0.02 M Na_2_S·9H_2_O in MeOH/water (1:1, v/v)) in succession. The deposition of PbS QD was repeated three times. TiO_2_/PbS films were then immersed in a Cd^2+^ (0.1 M Cd(CH_3_COO)_2_·2H_2_O in MeOH) and a S^2–;^ solution (0.1 M Na_2_S·9H_2_O in MeOH/water (1:1, v/v)) repeatedly for six times for CdS deposition. For the ZnS passivation layer, TiO_2_/QD heterostructured electrodes were additionally immersed in a Zn^2+^ (0.1 M Zn(CH_3_COO)_2_·2H_2_O in MeOH) and a S^2–^ solution (0.1 M Na_2_S·9H_2_O in MeOH/water (1:1, v/v)) twice repeatedly. Between each step, a cleaning process should be conducted for the production of high-quality QD films by rinsing the films with MeOH and DI water to remove impurities during the successive deposition. The immersion period for each step was 1 min per solution.

The fabrication method used to create the Cu*_x_*S counter electrode (CE) for PV cells was adopted from previous studies.^40,41^ Copper sulfides were deposited on FTO glass by the chemical bath deposition (CBD) method. A mixture of 0.1 M CuSO_4_ and Na_2_S_2_O_3_ aqueous solutions was used for the deposition of Cu*_x_*S films. Then, FTO was dipped in the solution at 70 °C for 3 h at pH 3, rinsed with DI water and EtOH, and dried using N_2_ gas. The as-prepared film was annealed at 130 °C for 30 min under ambient conditions. For PEC water splitting cells, a 2.5 cm × 2.5 cm commercial Pt plate was used.

### 2.3 Fabrication of PV Cells and PEC Water Splitting Cells

For PV cells, we employed the sandwich fabrication method using the Cu*_x_*S CE with two holes predrilled for electrolyte injection and the QD-photoanode. A 25 μm thick Surlyn film was placed between the two electrodes as a spacer, and the electrolyte composed of 2.0 M S, Na_2_S, and KCl in aqueous solution was injected into this space. The holes were then covered up using a Surlyn film and cover glass for finishing.

In PEC water splitting cells, a Pt plate (2.5 cm × 2.5 cm) and a Ag/AgCl (3 M KCl) electrode were used as the counter and the reference electrodes, respectively. The same photoanodes were used as PV cells. In the aqueous electrolyte, 0.25 M Na_2_S and 0.35 M Na_2_SO_3_ (pH 13) were utilized as hole scavengers. Sufficient N_2_ bubbling was conducted for over 30 min before every measurement. The characteristics of PEC water splitting cells for hydrogen generation were measured using a custom-built cell.

### 2.4 Characterization

#### 2.4.1 Morphology and Energy Level of TiO_2_

A transmission electron microscope (TEM) JEM 2100 F (JEOL, Japan) was used with the ZrO/W(100) electron gun. The specific surface areas and pore size distributions of TiO_2_ NPs were measured using Brunauer–Emmett–Teller (BET) and Barret–Joyner–Halenda (BJH) analyses with 3Flex (Micromeritics) using a N_2_ carrier gas. The crystal phase of TiO_2_ films coated on FTO substrates was measured by X-ray diffraction (XRD) using SmartLab (Rigaku) with Cu Kα radiation. Scanning electron microscope (SEM) images were measured using an FE-SEM (NOVA NANO SEM 450, FEI) at a 10.0 kV acceleration voltage and a field-free lens mode. To trace the energy level of TiO_2_ films, UV–vis absorption spectroscopy (V-670 UV/vis spectrophotometer, Jasco) was used for TiO_2_ without the scattering layer/FTO samples, and ultraviolet photoelectron spectroscopy (UPS, Thermo Fisher Scientific Co.) data were collected from a He I excitation source (21.2 eV). The UPS data were calibrated using the Fermi edge of Au. All samples for UPS were coated on a Si substrate.

#### 2.4.2 Measurement of Time-Resolved Photoluminescence (TRPL)

Time-resolved photoluminescence (TRPL) imaging was measured using an inverted-type scanning confocal microscope (MicroTime-200, Picoquant) with a 40× (air) objective at the Korea Basic Science Institute (KBSI) in Daegu Center (Republic of Korea). For an excitation source, a single-mode pulsed diode laser (470 nm with ∼30 ps pulse width and ∼10 μW laser power) was employed. The emission of each sample was collected using a single-photon avalanche diode (SPAD; PDM series, MPD). Using the time-tagged time-resolved (TTTR) data acquisition route, TRPL images (200 × 200 pixels) were recorded with 5 ms of the acquisition time for each pixel. Fittings of the PL decays were conducted by an exponential decay model using the following equation: *I*(*t*) = ∑*A_i_*e^((−*t*)/τ*i*)^, where *I*(*t*) is the time-dependent PL intensity, *A* is the amplitude, and τ is the PL lifetime.^42^

#### 2.4.3 Measurement of the Current–Voltage (*J*–*V*) Curve and Incident Photon-to-Current Conversion Efficiencies (IPCEs)

A Keithley (Model 2400) source meter and a solar simulator with a 300 W Xenon arc-lamp (Newport) were used for the measurement of the *J*–*V* characteristics of photoelectrochemical devices. The IPCEs of PV cells were measured using a QEX7 system (PV Measurements, Inc.). To avoid overestimating the power conversion efficiency (PCE) and the photocurrent density, masks (0.18 and 1.33 cm^2^ active areas) were placed on the front side of the photoanodes.

#### 2.4.4 Electrochemical Analyses

To estimate the density of surface states in the TiO_2_ films with and without SPL, electrochemical methods of cyclic voltammetry (CV) and impedance spectroscopy (IS) were used with a three-electrode system and an aqueous electrolyte consisting of 0.25 M Na_2_S and 0.35 M Na_2_SO_3_, which was the same composition used for PEC water splitting cells under dark conditions. Because of the basic pH of the electrolyte in the PEC water splitting cells (pH 13), a reversible hydrogen electrode (RHE) should be used as the reference electrode via correction of the Ag/AgCl reference electrode potential using the equation *V*_RHE_ = *V*_Ag/AgCl_ + 0.197 + pH × 0.059.^43^ The results of chemical capacitance (*C*_μ_) and recombination resistance (*R*_rec_) with the TiO_2_/PbS-CdS QD photoanodes were extracted from IS measurements through the simplified equivalent circuit in the dark for PEC water splitting cells.^34^ Different frequency ranges were applied in PV cells (1 MHz–10 mHz) and PEC water splitting cells (400 kHz–10 mHz) based on previous reports.^34,39^ The film conductivity (σ) was directly calculated from the charge transport resistance (*R*_t_) of the films, which was also analyzed by the IS measurements with the simplified equivalent circuit under dark conditions using the equation σ = *L*/*R*_t_*A*(1 – *p*_o_), where *L* is the film thickness, *A* is the film area, and *p*_o_ is the film porosity.^44^ The same quasi-Fermi levels in the TiO_2_ films for both *R*_t_ and *R*_rec_ were used to obtain a correct η_cc_ at 1 sun illumination.^45^ The equivalent conduction band potential (*V*_ecb_) was used for the IS parameters (*R*_t_ and *R*_rec_) and calculation of η_cc_. Here, *V*_ecb_ = *V*_F_ – Δ*E*_c_/*q*, where *V*_F_ is the corrected Fermi voltage, which is the actual potential applied to the photoanode without the voltage drops from the counter electrode and the series resistance from the applied potential, and Δ*E*_c_ is the shift in the CB with respect to the reference *E*_c,ref_; Δ*E*_c_ = *E*_c_ – *E*_c,ref_.^46^ For PV cells, IS has been frequently used to investigate the interfacial characteristics of charge accumulation, transport, and recombination using a diffusion–recombination equivalent circuit model.^47^

## 3 Results and Discussion

### 3.1 Morphological and Structural Characteristics

**Figure 2:**
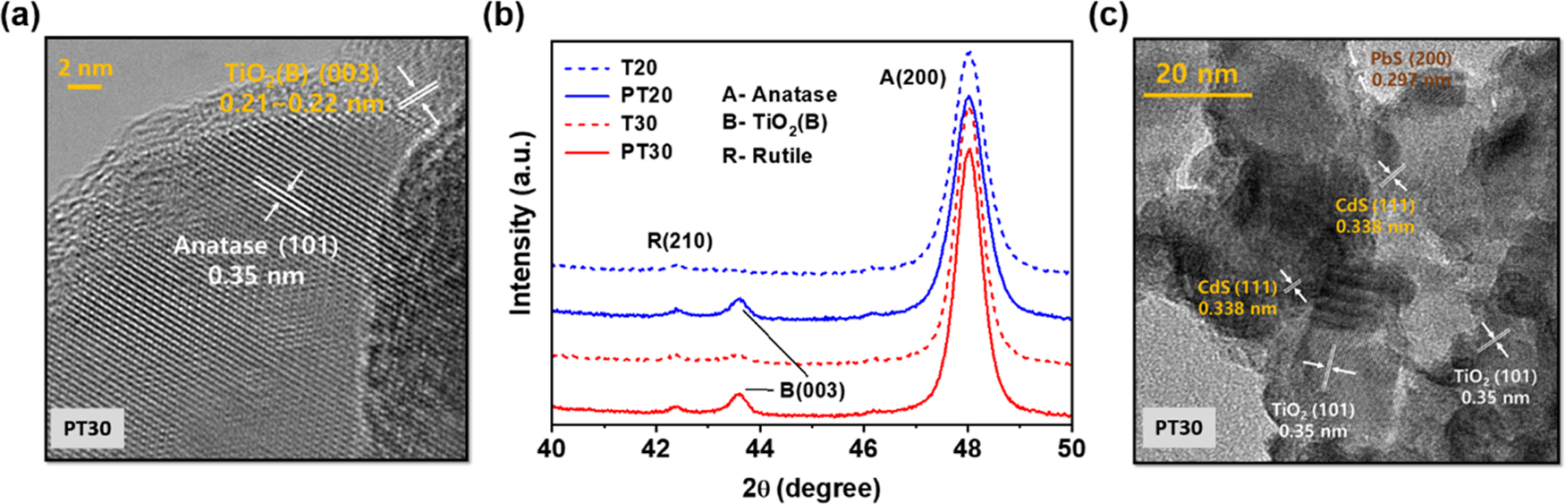
(a) HR-TEM image of PT30 with typical lattice distances of anatase (101), rutile (210), and TiO_2_(B) (003). (b) XRD patterns of TiO_2_ films indicating the peaks for anatase A (200) at 2θ = 48.0°, rutile R (210) at 2θ = 42.5°, and TiO_2_(B)(003) at 2θ = 43.5°. (c) TEM images with typical lattice distances of PbS(200) and CdS(111) QDs deposited on PT30.

Deposition of a surface passivation layer (SPL) by thermal hydrolysis of TiCl_4_ led to clear morphological changes in mesoporous TiO_2_ films, as shown in Figure S1a. Hereafter, mesoporous TiO_2_ layers created with NPs having 20 and 30 nm particle diameters are referred to as T20 and T30, and the corresponding ones with SPLs are denoted as PT20 and PT30, respectively. An SPL whose thickness ranged from 2 to 5 nm was identified at the surface of the mesoporous TiO_2_ films by TEM, high-resolution TEM (HR-TEM), and X-ray photoelectron spectroscopy (XPS) (Figures 2a and S1). In Figure 2a, the HR-TEM image of PT30 clearly indicates the effective growth of the SPL on the surface of TiO_2_ NPs. The highly textured atomic structure represents the (101) plane of the anatase phase of the TiO_2_ NP, and the corresponding *d*-spacing is 0.35 nm. On the top of the anatase surface, a distinct crystalline layer is formed with a comparable narrow *d*-spacing of 0.21–0.22 nm, attributed to the (003) plane of TiO_2_(B). Interestingly, XRD patterns (Figures 2b and S2) of PT20 and PT30 showed a dual-phase (anatase and TiO_2_(B)) nanocrystalline structure with polygonal plate- and needle-shaped crystallites, respectively, whereas only anatase crystals were obtained for T20 and T30 without the SPL (Figure S2). Here, a peak at 2θ = 48.0° corresponds to the anatase in the (200) direction, and another peak at 2θ = 43.5° (Figure 2b) is identified as the (003) plane of the polymorph TiO_2_(B) structure.^48,49^Figure 2a also clearly shows the interplanar distance of the TiO_2_(B) structure (0.21–0.22 nm) at the (003) plane, as confirmed by JCPDS no. 46-1237 in the SPL of PT30.^49^ TEM and XRD data suggested that the SPL created a distinct crystal structure of the TiO_2_(B) phase on the TiO_2_ films, which resulted in morphological and structural changes and a corresponding change in electron transport properties. However, further analyses were not carried out to reveal the characteristics of TiO_2_(B), such as a detailed growth mechanism and physical properties, since it was out of the scope of the current work.

Concerning the effects of the morphological changes on the SPL, BET and BJH data were found to correlate well with the SEM top-view images, indicating that there is a decrease in the pore size, pore volume, and surface area of the SPL (see Table 1 and Figures S3 and S4). For instance, the porosities (*p*_o_) of 58 and 66% for T20 and T30 were reduced to 43 and 65% for PT20 and PT30, respectively.

**Table 1:** Results of Multipoint BET and BJH Analyses with N_2_ Gas Adsorption and Desorption to Characterize the Surface Properties of Mesoporous TiO_2_ Films with/without SPL

samples	surface area (m^2^/g)	pore volume (cm^3^/g)	pore size (nm)	aporosity (%)	coordination number
T20	90.66	0.35	15.41	58	4.21
PT20	84.60	0.20	9.27	43	5.99
T30	71.64	0.50	30.95	66	3.54
PT30	70.00	0.47	26.87	65	3.63

a Porosity (*p*_o_) is calculated from the pore volume results using the equation *p*_o_ = *V*_p_/(1/ρ – *V*_p_), where *V*_p_ is the cumulative specific pore volume and 1/ρ is the reciprocal of the density of TiO_2_ (0.257 cm^3^/g).^30^

The average coordination number of TiO_2_ NPs (*N*), representing the average number of interconnections between TiO_2_ per particle, is readily calculated using the equation *N* = 3.08/*p*_o_ – 1.13, with *p*_o_ being the porosity,^50^ as listed in Table 1. *N* increased as the NP size decreased. Interestingly, the formation of the SPL notably resulted in an increase in *N*, particularly for PT20, suggesting a better architecture for electron transport.

PbS and CdS QDs were deposited on mesoporous TiO_2_ films to prepare heterostructured photoanodes for TiO_2_/PbS-CdS QDs by a SILAR method (see XRD and HR-TEM images of Figures S5 and S6). The deposition method is detailed in the Experimental Section. PbS and CdS QDs featuring particles 2–3 and 5–7 nm in diameter, respectively (Figure 2c and TEM and energy-dispersive X-ray spectrometry (EDX) images of Figure S7), were evenly distributed on the surface of TiO_2_ films (Figure S8). The surface area of TiO_2_ mesoporous films showed little difference after SPL deposition (Table 1), which would have a negligible influence on the surface coverage of QDs on TiO_2_ films. Atomic ratios of PbS and CdS QDs on the TiO_2_ films measured by XPS support the surface area results (Figure S9), suggesting that the surface fraction of deposited QDs on TiO_2_ films with the SPL would be slightly lower than that of neat samples without the SPL.

### 3.2 Photoelectrochemical Performances of PV Cells and PEC Water Splitting Cells

**Figure 3:**
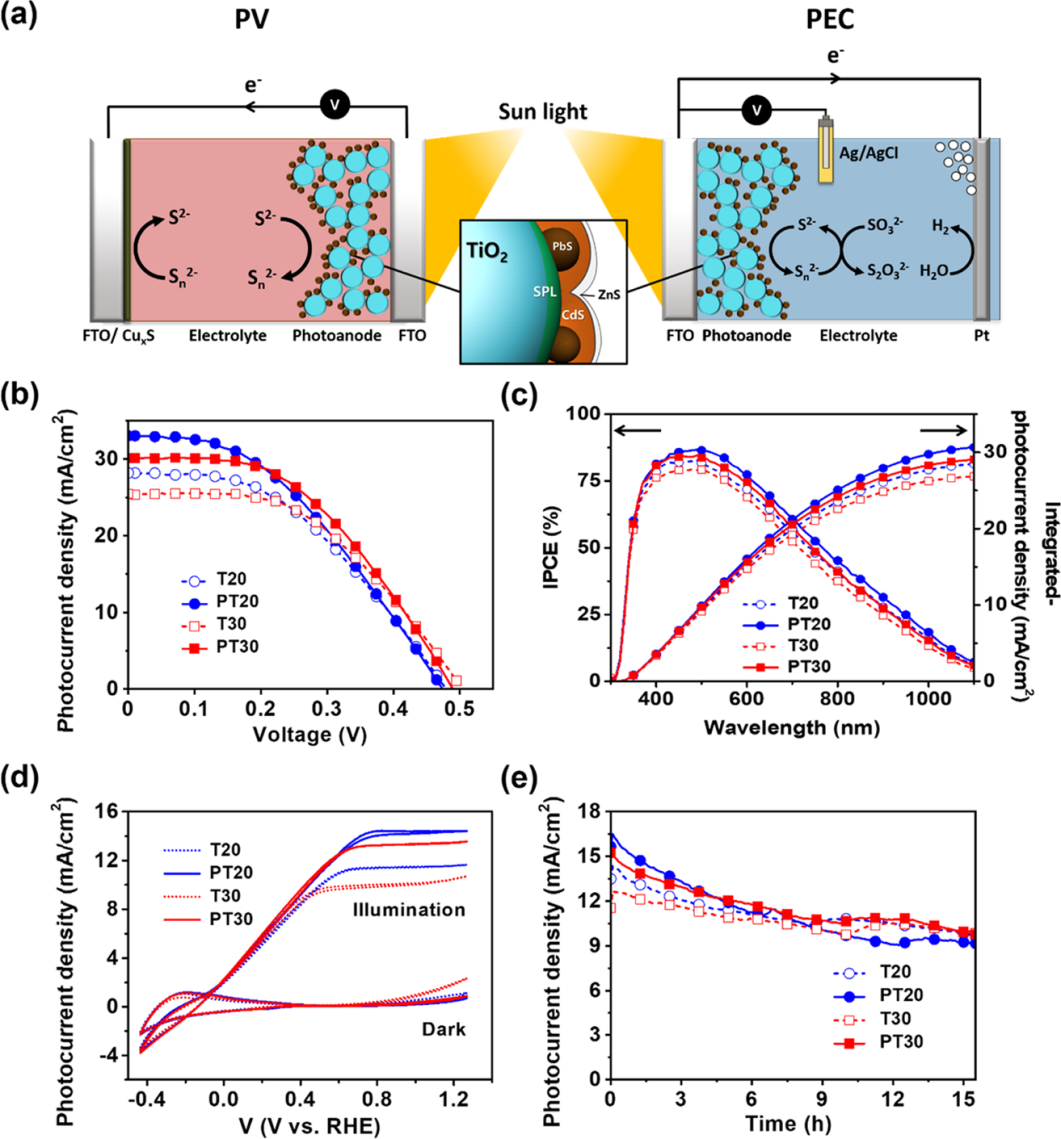
(a) Schematic illustrations of the structures and (b–e) performances of PV cells and PEC water splitting cells. The arrows in (a) indicate the charge transfer reactions between electrodes and electrolytes. Based on the same structure of both photoanodes for oxidizing sulfide (S^2–^) to polysulfide (S*_n_*^2–^), Cu*_x_*S CE regenerates the polysulfide at the PV cell, and the Pt CE reduces H_2_ from H_2_O at the PEC water splitting cell. (b) *J*–*V* curves for PV cells, (c) IPCE and integrated *J*_sc_ for PV cells using two-electrode sandwich cells, (d) *J*–*V* curves for PEC water splitting cells in the dark and under 1 sun conditions obtained by the three-electrode system with an Ag/AgCl reference electrode, and (e) chronoamperometric test for the long-term stability of PEC water splitting cells. All photocurrent densities were measured at 1 sun illumination. TiO_2_/QD photoanodes of T20 (the blue empty circles and the dotted line), PT20 (the blue filled circles and the solid line), T30 (the red empty squares and the dotted line), and PT30 nm (the red filled squares and the solid line).

The mesoporous TiO_2_/PbS-CdS QD heterostructured photoanodes with/without the SPL were applied for both PV cells and PEC water splitting cells to evaluate their performances. A ZnS passivation layer was additionally deposited on the photoanodes to prevent photocorrosion and reduce recombination. Figure 3a depicts the device architectures of PV and PEC cells with mesoporous TiO_2_/PbS-CdS QD heterostructured photoanodes.

The *J*–*V* curves of PV cells with a 0.18 cm^2^ active area of the photoanodes using the two-electrode sandwich devices at 1 sun conditions are shown in Figure 3b, and their corresponding photovoltaic parameters are summarized in Table 2. The IPCE and the integrated photocurrent density from the IPCE spectra support the *J*–*V* results of PV cells (Figure 3c). The short-circuit current density (*J*_sc_) significantly increased in the presence of the SPL (about 20%) compared to those of the references (T20 and T30), showing a *J*_sc_ higher than 25 mA/cm^2^ (Table 2). In particular, PT20 showed a *J*_sc_ of 34.59 mA/cm^2^ at 1 sun condition (Figure S11), which is the highest value reported in PV cells with TiO_2_/QD photoanodes to date (Figure 1), whereas PT30 achieved the best PCE of PV cells (6.85%). The pore sizes of mesoporous TiO_2_ films can account for the different PCEs observed in the samples. As shown in Table 1, the pore size of PT20 decreased to 9.27 nm after SPL deposition from the 15.41 nm of T20, increasing the likelihood of pore blockage when the QDs are deposited on mesoporous TiO_2_ films. However, PT30 probably could maintain an adequate pore size to allow for electrolyte penetration even after the formation of the SPL. The small pore sizes of PT20 are also related to the large series resistance (*R*_s_) in the film, directly resulting in a decreased FF and a consequent decrease in the PCE.

**Table 2:** Photovoltaic Characteristics of PV Cells with TiO_2_ Films of Photoanodes for T20, PT20, T30, and PT30^a^

type of TiO_2_	*V*_oc_ (V)	*J*_sc_ (mA/cm^2^)	FF	PCE (%)
T20	0.48	28.36 ± 0.20	0.43	5.88 ± 0.03
PT20	0.47	33.67 ± 0.30	0.41	6.46 ± 0.02
T30	0.50	25.42 ± 0.25	0.49	6.19 ± 0.02
PT30	0.49	29.56 ± 0.49	0.47	6.83 ± 0.02

a The numbers indicate the average photovoltaic performances (*J*_sc_ and PEC) and standard deviations for five or six different cells (see Figure S10 for *J*–*V* curves).

The outstanding photocurrents obtained for PV cells motivated us to use the photoanode in PEC water splitting cells for solar H_2_ production. In comparison with PV cells, the performance of PEC water splitting photoanodes with a larger geometrical area (1.33 cm^2^) showed similar trends (e.g., an increase in *J*_sc_ upon introduction of a SPL). Additionally, the photocurrent densities of PEC water splitting cells (*J*_ph_) were higher than 10 mA/cm^2^ at 0.82 V RHE for all samples, tested in a three-electrode arrangement (Figure 3d). Impressively, the highest *J*_ph_ of 14.4 mA/cm^2^ at 0.82 V RHE was achieved with PT20. Note that the potential of 0.82 V RHE is based on all samples at a steady state. This is remarkable compared to previously reported values (see Table S1 for comparison). The steady-state, long-term stability of the photoanodes was tested (Figure 3e) at the same applied voltage (0.82 V RHE), showing relatively stable operation above 9 mA/cm^2^ for more than 15 h under a strong alkaline condition (pH 13). H_2_ generation was monitored by gas chromatography equipped with gas-enclosed PEC water splitting cells, and the corresponding current density profile was recorded at 1.4 V RHE under the same irradiation condition (1 sun) (a and b Figure S12b). The trend of H_2_ generation was in excellent agreement with that of the photocurrent density in both PV cells and PEC water splitting cells. The faradic efficiency was also enhanced when the TiO_2_ films were treated with a SPL (Figure S12c).

### 3.3 Effects of the SPL on Charge Injection Efficiency

To gain further mechanistic insights into the operation of the photoelectrodes used, light-harvesting efficiency (η_lh_), charge separation efficiency (η_sp_), and charge collection efficiency (η_cc_) were determined.^24^ η_sp_ must be divided into charge injection and regeneration efficiencies (η_inj_ and η_reg_, respectively), and η_reg_ is assumed to be unity for all cases since the regeneration rate of QDs by excess S^2–^ in the electrolyte is nearly 3–4 orders of magnitude faster than the electron transfer rate from TiO_2_ to oxidized QDs.^21,51^

**Figure 4:**
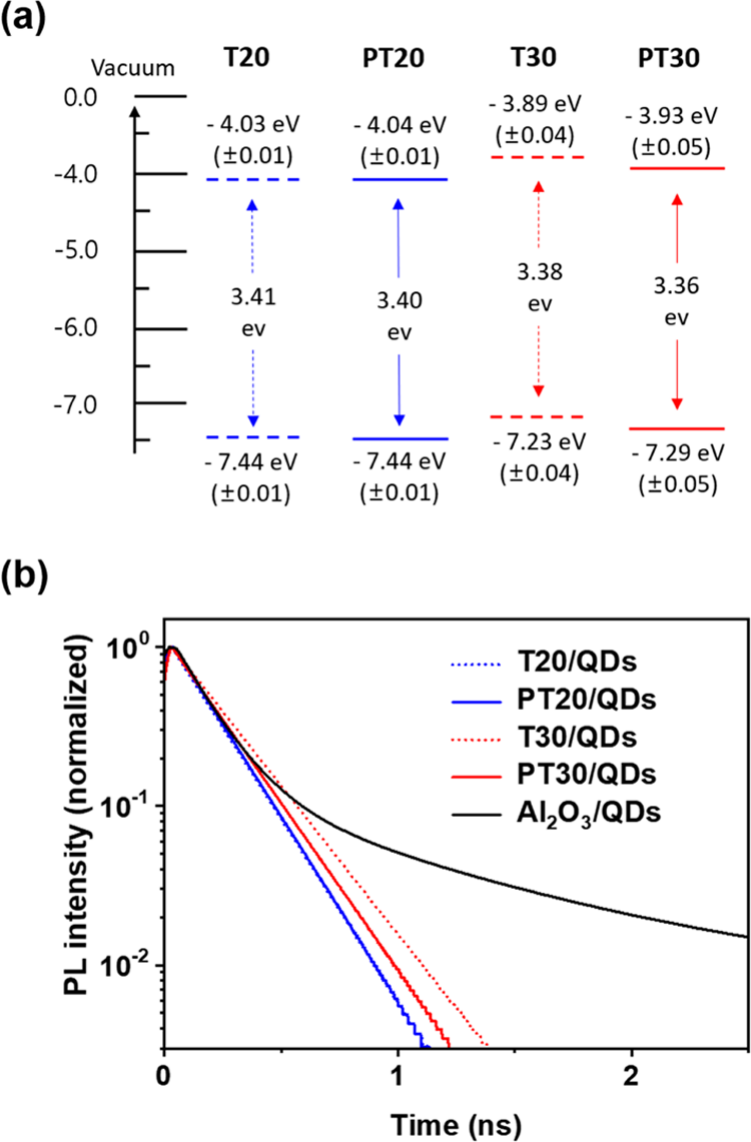
(a) Energy levels of TiO_2_ films obtained from the spectroscopic data of T20 and T30 and of PT20 and PT30. (b) TRPL emission decay spectra of TiO_2_/PbS-CdS QD heterostructured photoanodes measured at 500 nm. The Al_2_O_3_/QD film (black line) was used as the control electrode.

Consequently, the effects of the SPL on η_inj_ were examined following the Marcus theory.^52^ We considered the energy levels of TiO_2_ films determining the reaction free energy for the injection rate (Δ*G*^0^) from the CB of QDs (*E*_c_QD_) to the CB of TiO_2_ (*E*_c_TiO2_) films: Δ*G*^0^ = *E*_c_QD_ – *E*_c_TiO_2__. Based on the results of XRD and XPS for QDs (Figures S5 and S9), we assume that *E*_c_QD_ is the same in all samples. In other words, the relative Δ*G*^0^ value is solely determined by *E*_c_ TiO_2__. In this regard, we determined *E*_c_TiO_2__ first, as shown in Figure 4a, using the Tauc plots from UV–vis absorption spectra and UPS valence band (VB) spectra (Figure S13). These results suggest that the SPL slightly decreases the optical band gap (*E*_g_) of TiO_2_ films along with the CB energy level. PT20 (*E*_g_ = 3.40 eV) showed little difference from T20 (3.41 eV) in terms of band gap, while PT30 showed a larger change in the CB. The results also showed that Δ*G*^0^ increased in the following order: T30 < PT30 < T20 ∼ PT20.

Figure 4b shows the emission decay spectra of TRPL for QDs on TiO_2_ and QDs on Al_2_O_3_ as a reference, from which all electron injection parameters can be obtained.^53^Table 3 lists the electron injection parameters of PL lifetime (τ_PL_), electron injection rate constant (*k*_inj_), and electron injection efficiency (η_inj_). The results confirm that both *k*_inj_ and η_inj_ are consistent with Δ*G*^0^ in all TiO_2_/QD heterostructured photoanodes, suggesting their strong correlation. Even though a change in η_inj_ was observed, it was very small. This is different from the large increase in η_inj_ typically observed when an SPL was introduced by TiCl_4_ treatment in TiO_2_/dye photoanodes.^24^ Therefore, the reason for the above results is more likely due to electron injection from PbS QDs to the TiO_2_ film in the ultrafast range of femtoseconds.^54^

**Table 3:** Calculated PL Lifetime (τ_PL_), Electron Injection Rate Constant (*k*_inj_), and Electron Injection Efficiency (η_inj_) by the TRPL Emission Decay Spectra of TiO_2_/PbS-CdS QD Heterostructured Photoanodes^a^

photoanode semiconductors	τ_PL_ (ns)	b*k*_inj_ (10^9^/s)	cη_inj_ (%)
T20	0.189	5.11	96.62
PT20	0.188	5.14	96.64
T30	0.232	4.13	95.86
PT30	0.207	4.65	96.30
Al_2_O_3_	5.601		

a Al_2_O_3_/QDs were used for the control cell.

b τ_PL_ was calculated from the rate for electron injection by *k*_inj_ = 1/τ_titania_ – 1/τ_alumina_, where τ_titania_ and τ_alumina_ are the emission lifetimes of TiO_2_/QDs and Al_2_O_3_/QDs as the control cell, respectively.^42^

c η_inj_ was estimated through η_inj_ = *k*_inj_/(*k*_inj_ + *k*_non_), where *k*_non_ is the noninjection rate constant, and *k*_non_ = 1/τ_alumina_.^42^

### 3.4 Increases in the Surface State Concentration and the Charge Collection Efficiency

**Figure 5:**
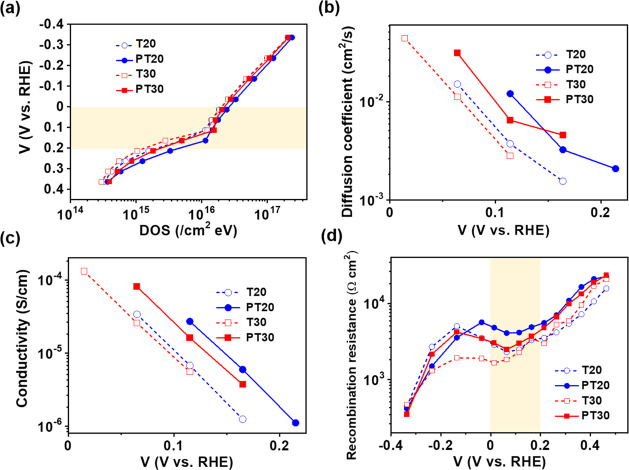
Several electrochemical parameters for charge accumulation and transport properties in the TiO_2_ films. (a) Calculated DOS of TiO_2_ films in the dark as a function of potential from chemical capacitance (*C*_μ_) below the CB. The yellow marked potential region indicates the peaks at 0–0.2 V, supporting the presence of surface states. (b) Chemical diffusion coefficient (*D_n_*), (c) electron conductivity (σ) calculated by charge transport resistance (*R*_t_) of the TiO_2_ films in the dark, and potentials for (b) and (c) are marked in yellow in (a). (d) Recombination resistance (*R*_rec_) of PEC water splitting cells with TiO_2_/PbS-CdS QD heterostructured photoanodes as a function of potential in the dark. The IS measurements were used to extract the parameters with the simplified equivalent circuit for transport, chemical capacitance, and charge transfer using a three-electrode system. T20 (blue empty circles), PT20 (blue filled circles), T30 (red empty squares), and PT30 nm (red filled squares).

It is well-known that surface states modified by the presence of an SPL play a key role in the energetic and kinetic properties for charge transport and transfer reactions, which are directly related to η_cc_.^34^ To estimate the density of surface states in the TiO_2_ films, chemical capacitance (*C*_μ_) was measured by cyclic voltammetry (CV) and electrochemical impedance spectroscopy (IS) in a PEC cell (Figure S14). Figure 5a shows the density of trap states (DOS) estimated from the *C*_μ_ of TiO_2_ films as a function of applied potential. The DOS peaks at 0–0.2 V RHE showed Gaussian behavior (marked as the yellow area in Figure 5a), which has been interpreted as a reversible filling of surface states located in traps below the CB of TiO_2_.^34,35,55^ The DOS increased after the deposition of the SPL (PT20 and PT30), suggesting that this layer increased the density of surface states on the TiO_2_ films.

The impact of the SPL on the energy distribution of TiO_2_ films directly correlates to the change of the chemical diffusion coefficient (*D_n_*). When a diffusion coefficient depends on *E*_F_, it is referred to as the “chemical” diffusion coefficient.^31,53,56^ Mesoporous nanostructured semiconductors featured charge transport within a broad distribution of localized states. Consequently, the change in electrochemical potential (Fermi energy level, *E*_F_) of charges modified the occupation of localized states, impacting their diffusion. Therefore, *D_n_* can be affected by both the energy distributions of nanostructured semiconductors and morphology. Figure 5b shows *D_n_* as a function of applied potential for TiO_2_ films. *D_n_* of all samples increased with negative potential, indicating that the transport mechanism follows the multiple trapping/detrapping transport model.^56^ Interestingly, *D_n_* exhibited the same trends as DOS and increased as T30 < T20 < PT30 < PT20 in the potential range of 0–0.1 V RHE. These results provide strong evidence that the surface states in the TiO_2_ film introduced by the SPL have a direct correlation with *D_n_*. Since the energy level of the surface states of the TiO_2_ films is located at the effective range of conduction (about 0.5–1.0 eV below the CB),^56^ we conclude that *D_n_* increases due to the increase in the density of surface states.

On the other hand, morphological changes such as porosity (*p*_o_), coordination number (*N*), and particle size induced by the SPL can also affect the intrinsic diffusion properties of TiO_2_ films. As expected, *D_n_* increases with an increase in *N* and a decrease in *p*_o_ during SPL formation.^57^ Regarding the particle size effect, we expected that the small TiO_2_ NPs would have a low *D_n_* primarily due to the increased grain boundary area.^30^ However, Figure 5b shows that the small TiO_2_ NPs have a high *D_n_* instead. Therefore, we conclude that both *p*_o_ and *N* have a stronger influence on *D_n_* compared to the particle size. In any case, surface states and particle sizes may be related.^58^

Based on the multiple trapping/detrapping transport model for charge transport in mesoporous nanostructured semiconductors and the generalized Einstein relation, conductivity (σ) can be expressed by the following equation
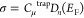
1Here, *C*_μ_^trap^ is the trap state capacitance, and *D_n_*(*E*_F_) is the electron diffusion coefficient.^53^

If the density of trap states can produce localized electrons, the DOS in the band gap is associated with the trap state capacitance. Therefore, the energetic distribution of the density of trap states (DOS = *g*(*E*_F_)) can be calculated by the equation *C*_μ_^trap^(*E*) = *qg*(*E*_F_), where *q* is the elemental charge.^35,47^ Clearly, the SPL increased the conductivity of TiO_2_ films through an increase in the density of surface states and morphological changes in the TiO_2_ film (Figure 5c).

Electron recombination is another important factor affected by the SPL via the density of surface states, significantly influencing charge collection. Figure 5d shows the evolution of recombination resistance (*R*_rec_) of heterostructured TiO_2_/PbS-CdS photoanodes with the applied potential. This resistance increased with the anodic (positive) potential. The valley at 0–0.2 eV is related to recombination through surface states.^34^ Interestingly, although the density of surface states was increased by the SPL, it efficiently blocked electron recombination. Furthermore, *R*_rec_ and σ followed similar trends and were increased by the SPL. The decreased dark currents in Figure 3d treated with the SPL supported well the recombination blocking effect of the SPL.

Both conductivity and recombination through the TiO_2_ films ultimately influence the charge collection efficiency, η_cc_, which in turn directly controlled the photocurrent densities (*J*_sc_ and *J*_ph_) of the photoelectrochemical devices. η_cc_ can be estimated by the equation
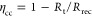
2where *R*_t_ is the transport resistance.^45,59^

**Figure 6:**
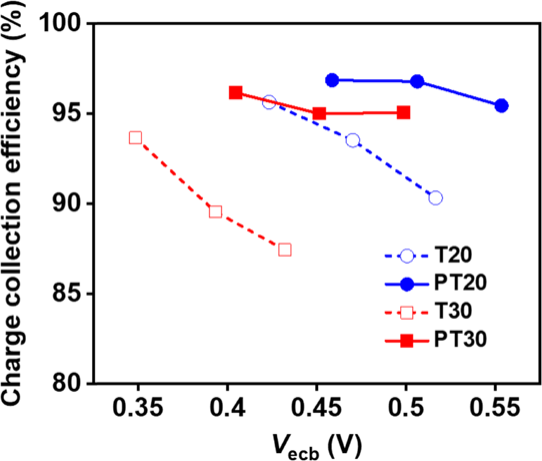
Charge collection efficiency (η_cc_) of PV cells with TiO_2_/PbS-CdS QD heterostructured photoanodes with T20 (blue empty circles), PT20 (blue filled circles), T30 (red empty squares), and PT30 nm (red filled squares) at 1 sun condition. Values were calculated using the results of *R*_t_ and *R*_rec_ as a function of *V*_ecb_ in PV cells (Figure S15).

Figure S15 shows the results of *R*_t_ and *R*_rec_ as a function of the equivalent conduction band potential, *V*_ecb_, in PV cells extracted from the IS measurements. Here, *V*_ecb_ can be obtained from *V*_ecb_ = *V*_F_ – Δ*E*_c_/*q* to compare the recombination resistance (*R*_rec_), where *V*_F_ is the corrected Fermi voltage excluding the influences of series resistance and the CE in the device and Δ*E*_c_ is the shift in the CB with respect to the reference *E*_c,ref_; Δ*E*_c_ = *E*_c_ – *E*_c,ref_.^46^ The calculated η_cc_ shown in Figure 6 increases with nearly the same trend as the conductivity and recombination resistance of the TiO_2_ films and the photocurrent densities of TiO_2_/PbS-CdS QD heterostructured photoanodes in both PV cells and PEC water splitting cells. Comparing the parameters for photocurrent densities such as the η_lh_ calculated via IPCE in Table S2, η_cc_, and η_inj_ values, we demonstrated that η_cc_ controls the photocurrent densities of PV cells with the TiO_2_ films. Consequently, high photocurrent densities greater than 30 mA/cm^2^ for PV cells with SPL samples (PT20 and PT30) were obtained primarily due to the enhancement of film conductivity, as well as the decrease in recombination kinetics induced by the SPL.

**Figure 7:**
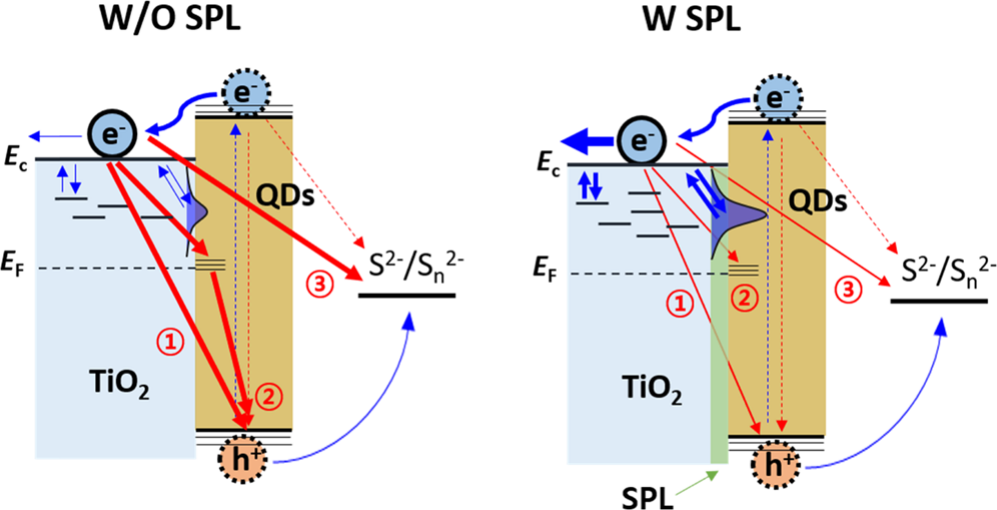
Schematic illustration of the effects of the SPL on the kinetics of the TiO_2_/QD heterostructured photoanode with the S^2–^/S*_n_*^2–^ electrolyte. The left and right sides indicate without (W/O) and with (W) an SPL, respectively. The blue arrows represent the charge flow pathway for the current and the red arrows represent recombination pathways of electrons under illumination.

Figure 7 summarizes the effects of the SPL on the photocurrent density of the TiO_2_/QD heterostructured photoanode.

- After light absorption of QDs, neither charge injection nor regeneration of oxidized QDs was significantly affected by the SPL. This may be partly related to the ultrafast electron injection rate of QDs caused by the direct deposition method.^54^
- The increased density of surface states induced by the SPL facilitated multiple trapping transport, which was demonstrated by the improvement of the chemical diffusion coefficient. The facilitated multiple trapping/detrapping transport along with morphological changes of the mesoporous TiO_2_ films (related to increased *N*) increased the electron conductivity of TiO_2_ films.
- Due to the different energy levels of CdS and PbS QDs, the electrons at the CB of TiO_2_ without an SPL can have multiple pathways for electron recombination through the TiO_2_/QD and TiO_2_/electrolyte interfaces such as (1) electron transfer to the long-lived holes at the VB of QDs, (2) QD trap-mediated electron transfer, and (3) electron transfer to the electrolyte. We demonstrated that the SPL can play a key role in decreasing the recombination kinetics at the TiO_2_/QD and TiO_2_/electrolyte interfaces. However, to confirm directly the effects of the SPL on (1) and (2), further experiments are required.

Since both η_inj_ and η_lh_ are almost unity at the TiO_2_/QD heterostructured photoanode, maximizing the photocurrent density by improving η_cc_ can be an effective strategy. A higher photocurrent density can be obtained by incorporating an SPL despite the slight loss of η_lh_ due to the decreased surface area (Table S2). As a result, the introduction of a SPL is a viable strategy to maximize η_cc_ and to optimize the photocurrent density by enhancing conductivity and blocking recombination at the TiO_2_/QD heterostructured photoanode.

## 4 Conclusions

We investigated the effect of a TiO_2_ SPL deposited on a mesoporous TiO_2_ film surface for TiO_2_/PbS-CdS QD heterostructured photoanodes for PV and water splitting PEC devices. Such a TiO_2_ SPL increased the density of surface states and also the coordination number of the TiO_2_ NPs, both leading to an increase in electron conductivity through the TiO_2_ film. The increase in electron conductivity in the TiO_2_ film is mostly due to the improvement of the chemical diffusion coefficient according to the multiple trapping/detrapping transport model. Concomitantly, the reduction of back electron transfers, known to be the conventional role of SPLs, also helps to increase η_cc_ in the TiO_2_ films. Therefore, the TiO_2_/PbS-CdS QD photoanodes showed a *J*_sc_ of 34.59 mA/cm^2^ in PV cells and a photocurrent density of 14.43 mA/cm^2^ at 0.82 V RHE in PEC water splitting cells. On the other hand, η_inj_, which mainly affects the photocurrent density in photoanodes, was not significantly different for devices with/without SPLs. The results could provide new directions and important milestones for the development of high-performance PEC devices with TiO_2_/QD heterostructured photoanodes.

## Supporting Information Available

TEM, XPS, XRD, BET, BJH, SEM, EDX, and conformal images of fluorescence lifetime for morphology and structure characteristics; *J*–*V* curve; IPCE of the PV cell for PEC performances; hydrogen generation amounts and faradic efficiency of PEC water splitting cells; UPS and UV–vis absorption spectra for energy levels of samples; electrochemical analysis results for surface states and charge collection; and previous reported PEC performances of metal oxide/QD heterostructured photoanodes for hydrogen generation and calculated light-harvesting efficiency (PDF)
