# Supplementary material for: Interfacial Engineering at Quantum Dot-Sensitized TiO2 Photoelectrodes for Ultrahigh Photocurrent Generation
Source: ACS Appl Mater Interfaces. 2021 Feb 1;13(5):6208–18. doi: 10.1021/acsami.0c19352 (PMC8576758; doi:10.1021/acsami.0c19352)
Supplement: Supplementary file 1 — am0c19352_si_001.pdf [file am0c19352_si_001.pdf]

Supporting Information

Interfacial Engineering at Quantum Dot-Sensitized TiO_2 Photoelectrodes for Ultrahigh Photocurrent Generation

Tea-Yon Kim, [‡a] Byung Su Kim,[‡b] Jong Gyu Oh,[c] Seul Chan Park,[b] Jaeyoung Jang,[c] Thomas W. Hamann,[a] Young Soo Kang,[d] Jin Ho Bang,*[e] Sixto Giménez,*[f] and Yong Soo Kang*[b]

[a]Department of Chemistry, Michigan State University, East Lansing, MI 48824-1322, United States

[b]Department of Energy Engineering and Center for Next Generation Dye-Sensitized Solar Cells, Hanyang University, Seoul 04763, Korea

[c]Department of Energy Engineering, Hanyang University, Seoul 04763, Korea

[d]Korea Center for Artificial Photosynthesis and Department of Chemistry, Sogang University, Seoul, 04107, Korea

[e]Department of Chemical and Molecular Engineering and Department of Applied Chemistry, Center for Bionano Intelligence Education and Research, Hanyang University, Ansan, Gyeonggi-do 15588, Korea

[f]Institute of Advanced Materials (INAM), Universitat Jaume I, Castelló 12006, Spain

1. Morphology and structure characteristics

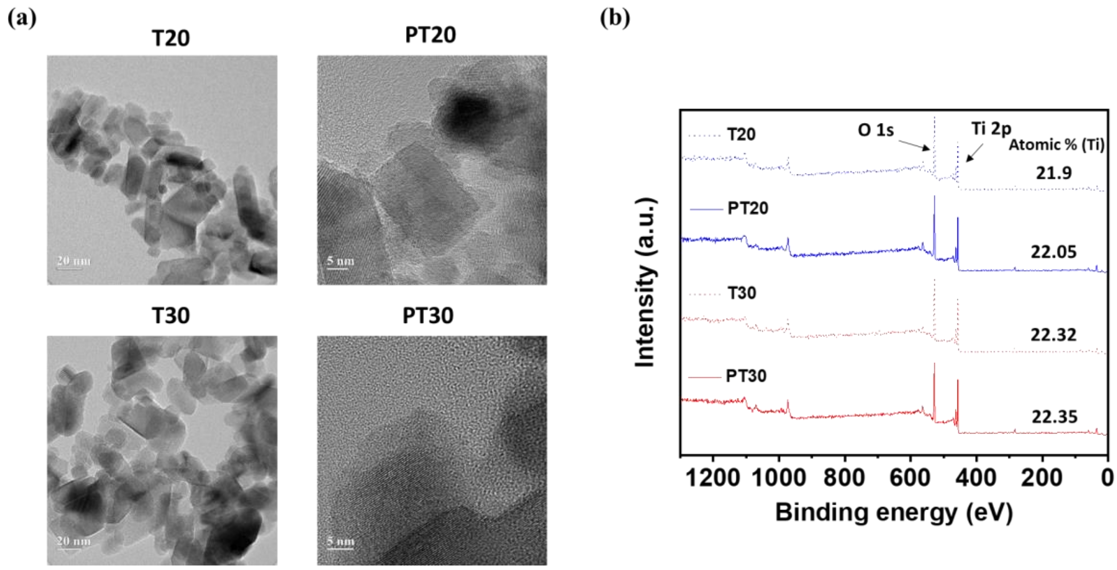

Figure S1. Formation of surface passivation layers (SPLs) on both 20 nm- and 30 nm-sized TiO_2 nanoparticles. (a) TEM and HR-TEM of both TiO_2 nanoparticles without and with SPL (T20, PT20, T30, and PT30). (b) XPS survey spectra and atomic ratios of Ti for each TiO_2 film.

The TEM images (Figure S1a) of T20 and T30 verified the successful deposition of SPL by hydrothermal $TiCl_4$ treatment on top of TiO_2 nanoparticles (NP). The atomic composition on each TiO_2 sample was analyzed by XPS (Figure S1b). In both cases, the atomic wt% of Ti was increased in the SPL-treated samples.

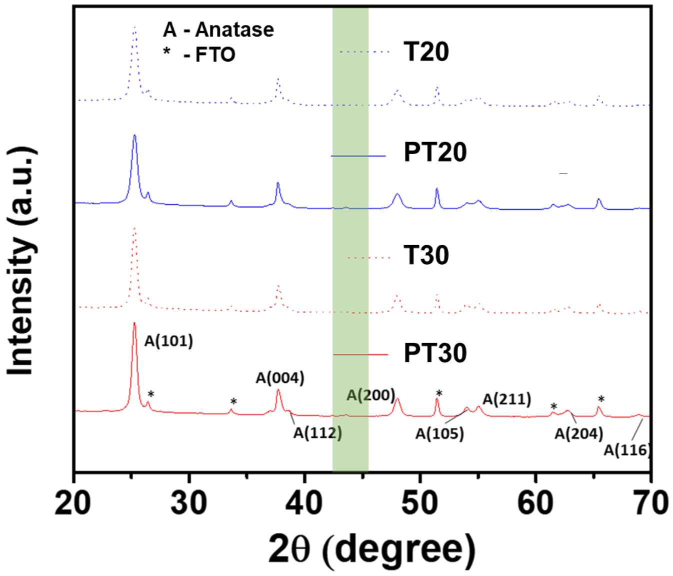

Figure S2. XRD patterns of TiO_2 films on FTO substrate. A and * denote XRD peaks for anatase and FTO, respectively. The new, small peak (2θ = 43.5 °, green region) indicates the TiO_2(B) structure of the (003) plane formed by hydrothermal $TiCl_4$ post-treatment as confirmed by JCPDS 46-1237. The green shaded area corresponds to Figure 2b.

The crystal structures of TiO_2 nanoparticles with and without SPL treatment were analyzed by XRD. Although the strong peaks indicate that the main body of TiO_2 consists of the anatase structure due to the hydrothermal $TiCl_4$ treatment, a new TiO_2(B) phase featured with the (003) plane (2θ = 43.5°) was observed for the NPs with SPL. In general, the thermal hydrolysis treatment of a precursor, such as $TiCl_4$, can produce the TiO_2(B) structure, and it has been reported that this structure is preserved even after sintering at the temperature range of 400 to 500 °C.[1-2] Compared to other TiO_2 polymorphs, the TiO_2(B) phase has larger interstitial sites inside the lattices, which promote intercalation of small ions like Li^+.[3-4]. Furthermore, due to the nature of thermal hydrolysis for the synthesis of TiO_2(B) phase, a high density of surface states exist in this phase,[5] which can also have a strong effect on electron transport in TiO_2 films.

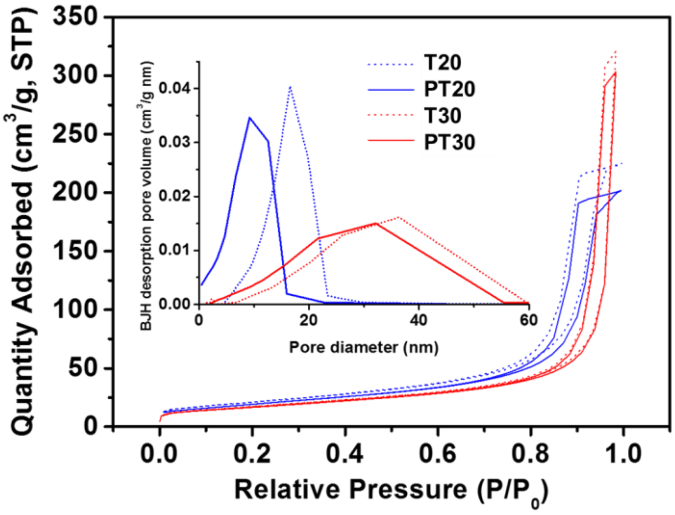

Figure S3. Brunauer–Emmett–Teller (BET) analysis for nitrogen adsorption/desorption isotherms. Inset shows Barret−Joyner−Halenda (BJH) pore size distribution plots of TiO_2 samples without the SPL (T20, T30; blue- and red-dot) and those with the SPL (PT20, PT30; blue- and red-line). The surface area and porosity of each sample were obtained from the N_2 adsorption-desorption and BJH pore size distribution experiments. The detailed values are listed in Table 1. As shown in Table 1, the P_o of T20 and T30 is well matched to the typical range of P_o in mesoporous, nanocrystalline TiO_2.[6]

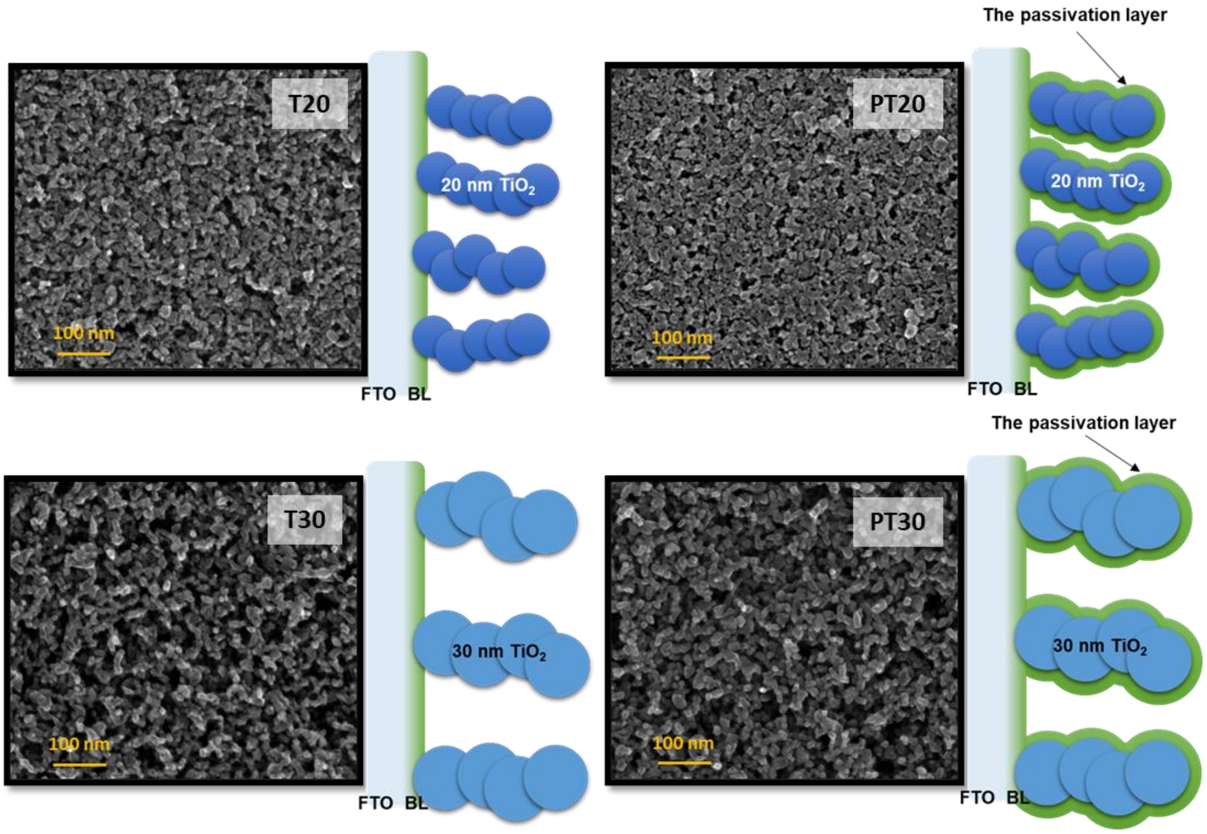

Figure S4. SEM images and schematic illustration of different sized TiO_2 NP mesoporous films with/without the SPL.

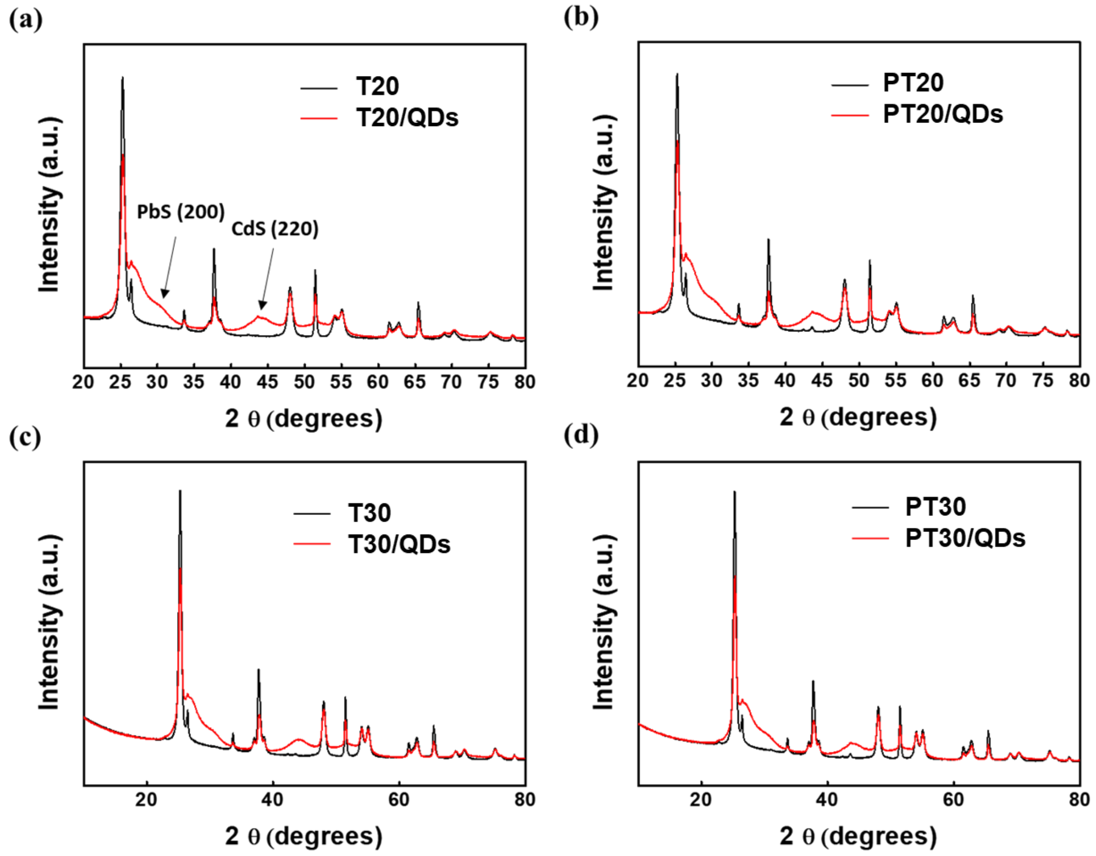

Figure S5. XRD patterns of PbS-CdS QDs deposited on (a) T20, (b) PT20, (c) T30, and (d) PT30.

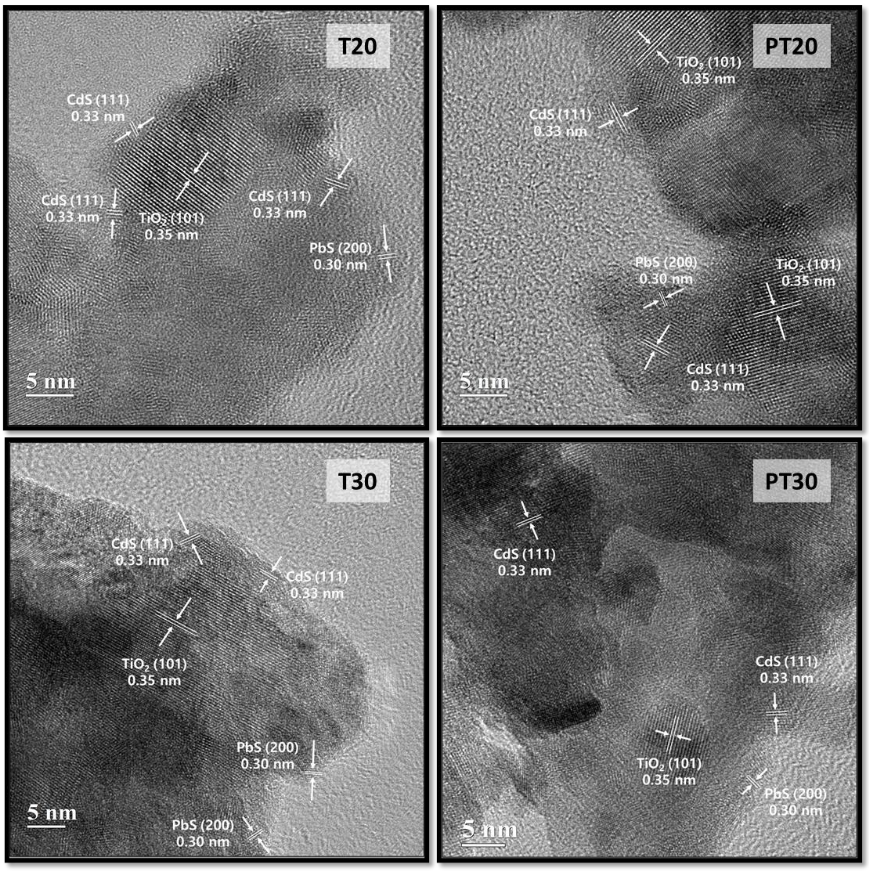

Figure S6. HR-TEM images of TiO_2/QDs photoanodes (T20, PT20, T30, and PT30). The lattice fringes observed in the images are the (101) planes of TiO_2, the (200) planes of PbS, and the (111) planes of CdS.

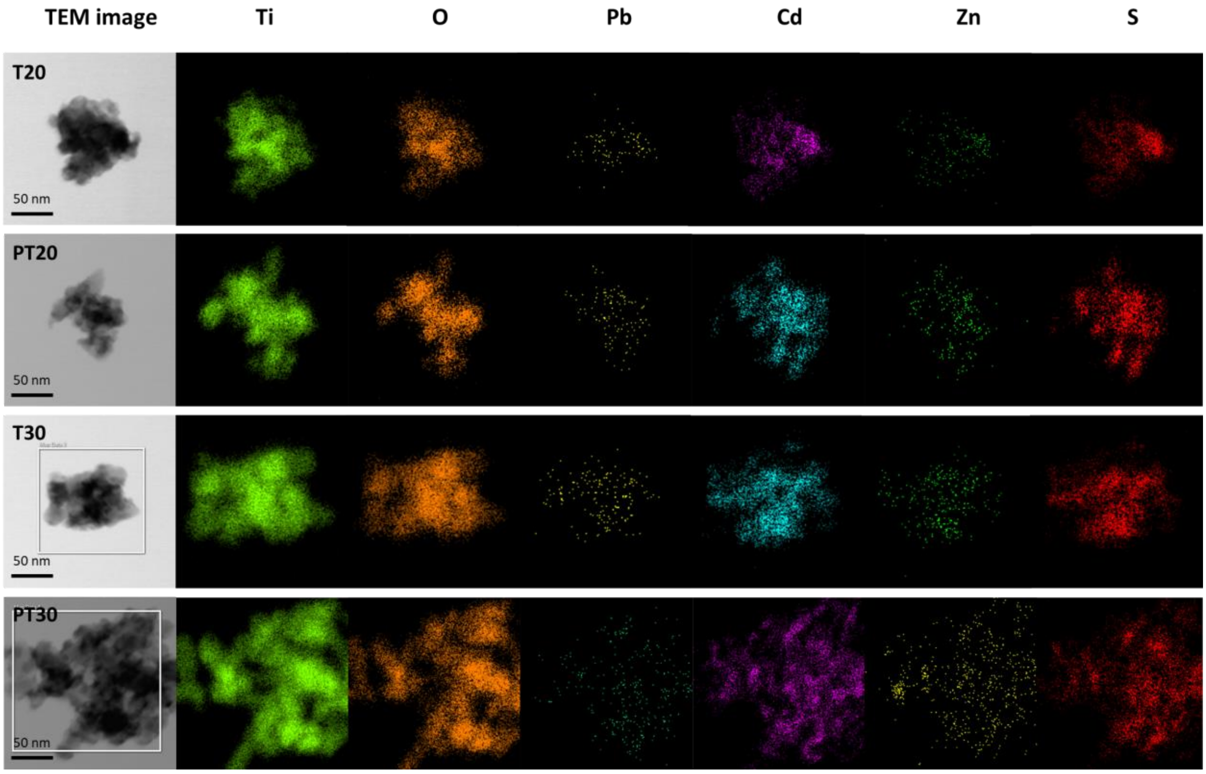

Figure S7. Energy-dispersive X-ray spectroscopy (EDX) images of TiO_2/PbS-CdS QD heterostructures from T20, PT20, T30, and PT30 samples. This result demonstrates a homogeneous coating of QDs on the TiO_2 surface for all samples.

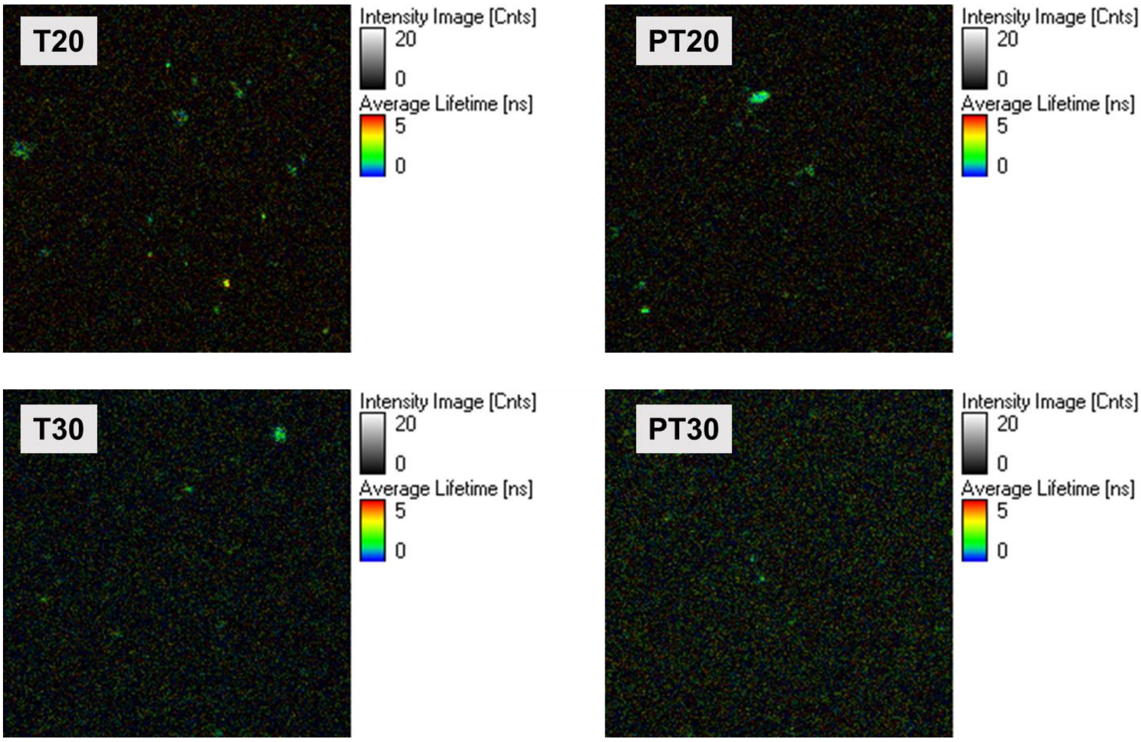

Figure S8. Conformal images of the fluorescence lifetime of PbS-CdS QDs deposited on TiO_2/QD heterostructured photoanodes. The conformal images (200×200 pixels) of the fluorescence lifetime for all PbS-CdS QDs deposited on the TiO_2 films confirmed a homogeneous coating. The color of each pixel indicates the average lifetime of electrons in the QDs before injection into TiO_2.

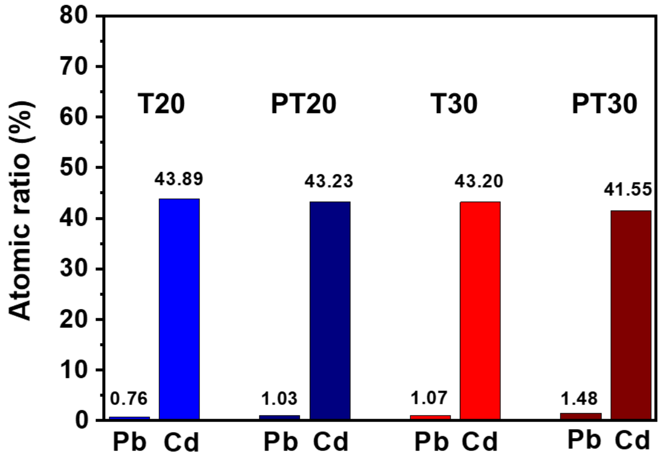

Figure S9. Atomic ratios of Pb and Cd in PbS and CdS QDs on TiO_2/QD heterostructured photoanodes measured by XPS.

2. Photoelectrochemical performances

2.1 J−V curves of PVs

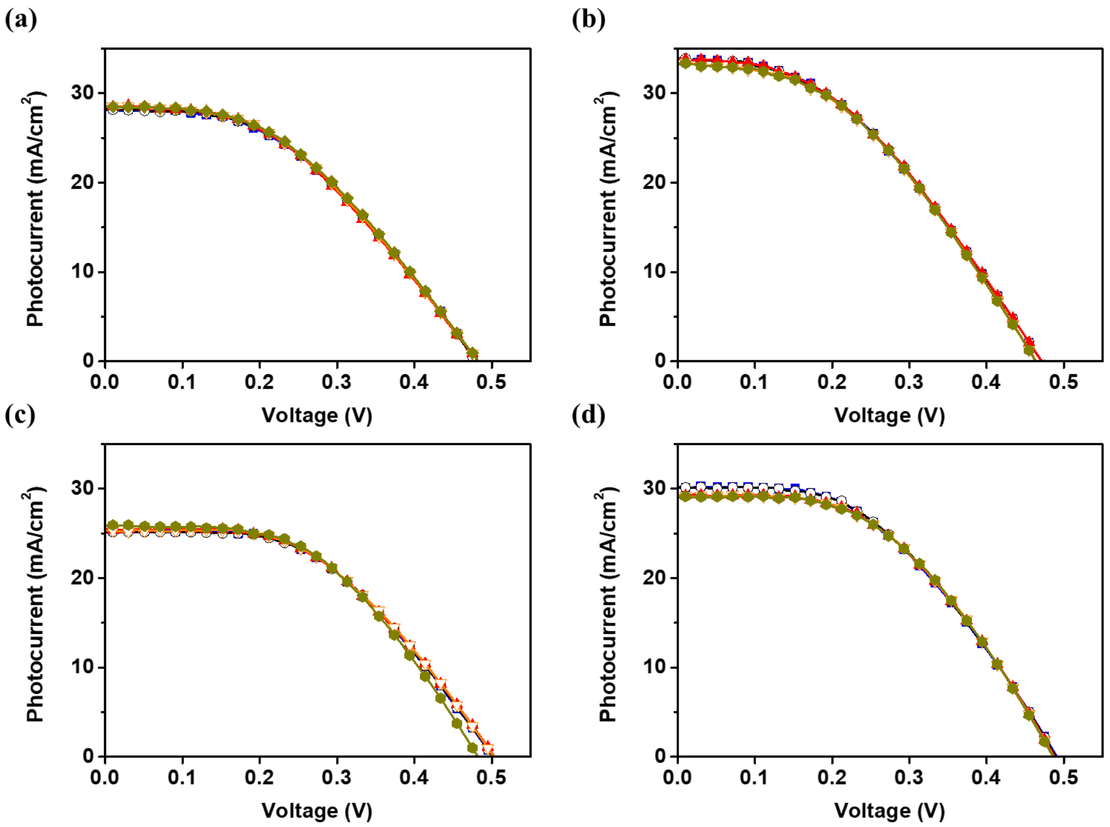

Figure S10. J−V curves of PVs with difference TiO_2 films: (a) P20, (b) PT20, (c) P30, and (d) PT30. The J−V curves were measured from five different cells to ensure reproducibility.

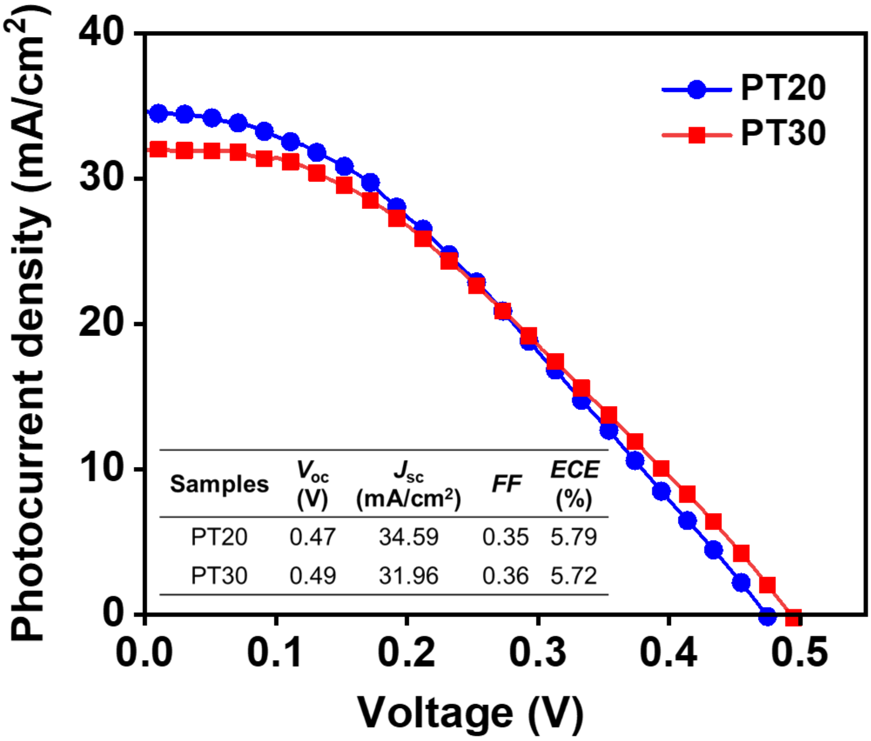

Figure S11. J−V curves of the best PVs with PT20 (blue circles) and PT30 (red squares) measured under 1 sun condition. The photovoltaic parameters are presented in the inset table.

2.2 IPCE measurements of PVs

The integrated J_{sc} according to IPCE had the same tendency as in the J_{sc} of $J-V$ curves but was slightly lower. One of the main reasons for this difference may be attributed to the absorption ability of PbS QDs up to 1600 nm that was not included in the IPCE results because the IPCE instrument uses a lamp that is capable of measuring only up to 1200 nm, unlike the light source used for the $J-V$ measurements (the xenon lamp can cover all wavelengths of light).[7]

2.3 PECs for hydrogen generation

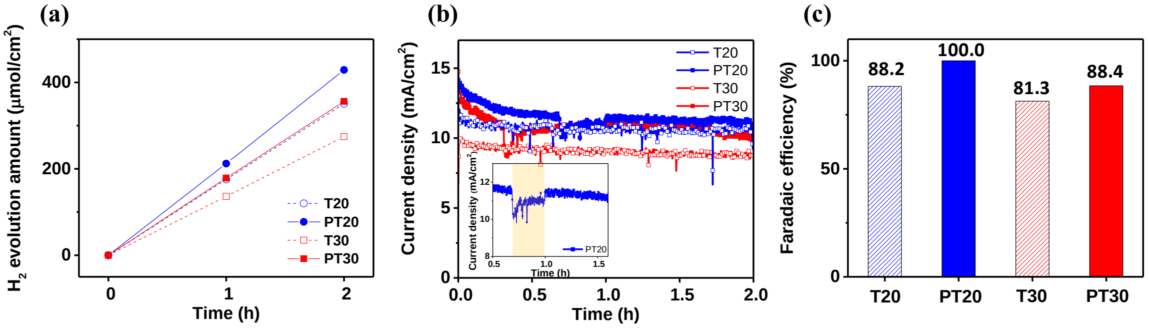

Figure S12. (a) Hydrogen generation of PECs with T20, PT20, T30, and PT30. (b) Current profiles vs. time measured at 1.4 V vs. RHE under 1 sun irradiation (inset for PT20). (c) Corresponding Faradaic efficiency of each PEC calculated from (a) and (b).

The amount of H_2 generation was measured using gas-enclosed PECs under 1 sun irradiation condition at 1.4 V RHE for 2 h, as shown in Figure S12a. Overall, the H_2 generation was much improved for TiO_2 films with SPL (PT20 and PT30) compared to neat TiO_2 films (T20 and T30). Notably, the largest amount of H_2 (428.9 μmol/cm^2) was obtained from the PT20. At the same time, chronoamperometric results were obtained at 1.4 V RHE during the hydrogen generation test (Figure S12b). To further estimate the conversion of photocurrent density to hydrogen generation, we evaluated the Faradaic efficiency (FE) of PECs (Figure S12c). The FEs of T20, T30, and PT30 were 88.2, 81.3, and 88.4%, respectively, which indicates that photo-generated electrons were consumed to produce hydrogen. The Faradaic efficiency was calculated using the equation below:[8]

$$\text{Faradaic efficiency (\%)} = \frac{2 \times mol\ of\ H_2 \times F\ (\frac{C}{mol})}{Charge\ passed\ through\ working\ electrode\ (C)} \times 100$$

We believe that the reason for the increased values of FE for the SPL treated films is related to

the enhanced stability of the samples, which is directly connected to the inhibition of detrimental side reactions at the photoanode.

3. Charge injection

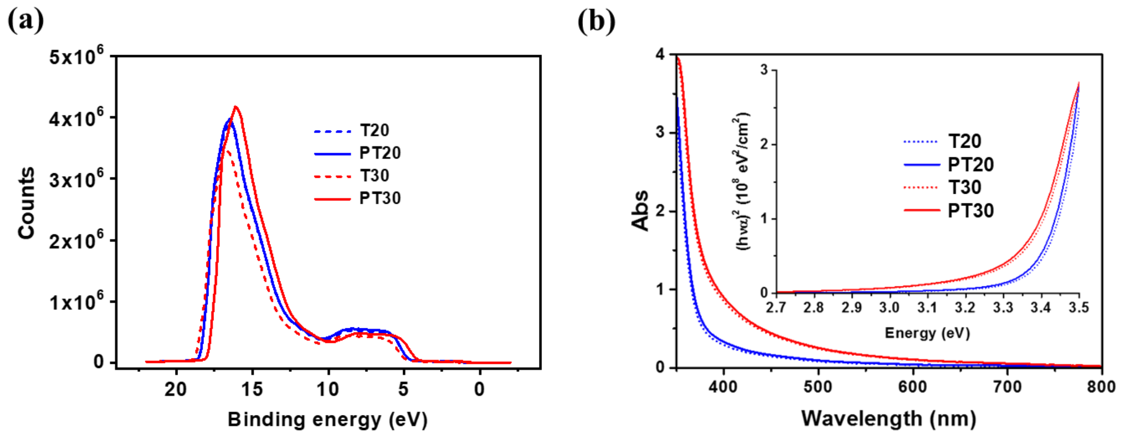

Figure S13. Estimation of energy levels of all TiO_2 films. (a) ultraviolet photoelectron spectrum and (b) UV-vis absorption spectrum and the Tauc plots (inset) for the determination of optical bandgap.

4. Surface states and charge collection

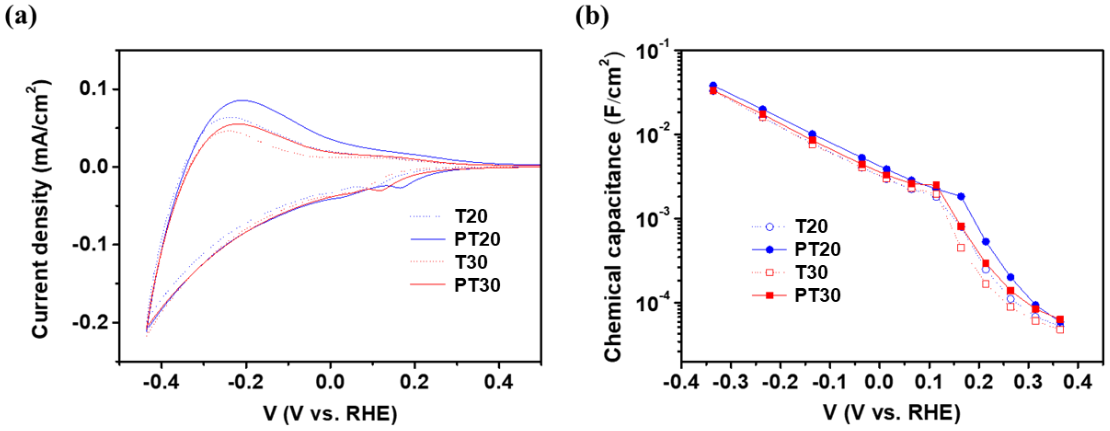

Figure S14. Estimation of density of surface state (DOS) in the TiO_2 films with and without the SPL from electrochemical methods under dark conditions using a three-electrode configuration with aqueous electrolytes of 0.25 M Na_2S and 0.35 M Na_2SO_3 (RE: Ag/AgCl and CE: Pt): (a) cyclic voltammetry curves of the TiO_2 films and (b) their chemical capacitance (C_μ) obtained from impedance measurements as a function of potential.

As shown in Figure S14a, all TiO_2 films show peaks at 0–0.2 V vs. RHE in the cyclic voltammetry (CV) results. Figure S14b indicates the chemical capacitance (C_μ) as a function of potential measured by impedance spectroscopy (IS). The C_μ was analyzed using a simplified equivalent circuit accounting for transport, chemical capacitance, and charge transfer of electrons, as in a previous study.[9] The peaks found in the C_μ plots have been explained as reversible filling of surface states below the conduction band (CB) of TiO_2.[9] These potential scans of the capacitance in the CV and IS allowed direct measurement of the surface state of a semiconductor, which shows Gaussian behavior.[9-10] All the surface states below the CB are involved in the trap states, and the surface state capacitance (C_μ^{ss}) is assumed to be equal to the trap state capacitance (C_μ^{trap}) in this work.[11]

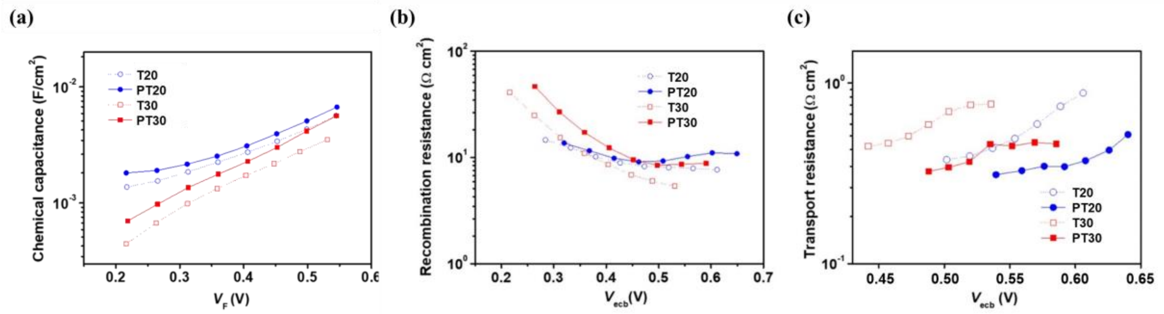

Figure S15. Energetic and kinetic properties of PVs with TiO_2/PbS-CdS QD heterostructured photoanodes calculated from IS measurement under 1 sun condition. (a) Chemical capacitance (C_μ) with respect to Fermi level voltage (V_F, removing the influences of series resistance and CE). (b) Recombination resistance (R_{rec}) and (c) selected transport resistance (R_t) as a function of equivalent common conduction band potential (V_{ecb}). To extract C_μ, R_{rec}, and R_t from the IS results, the diffusion-recombination model for mesoporous PVs was used as the equivalent circuit for data fitting.[12]

The results of V_{oc} suggest a reason for higher *ECE* values in PVs with T30 and PT30. Figure 3b and Table 2 in the main article showed that the V_{oc} of PVs increased in the order of PT20 < T20 < PT30 < T30. The V_{oc} of the mesoporous solar cells depends on the quasi-Fermi level ${}_nE_F$ of electrons in TiO_2 films when the electrolyte redox potential (E_{redox}) was constant under illumination, $V_{oc} = ({}_nE_F - E_{redox})/q$.[13] Because the ${}_nE_F$ of TiO_2 films is determined by the energy level of the TiO_2 conduction band (CB, E_c), V_{oc} can be expressed by the correlation of $V_{oc} \propto E_c$ at fixed E_{redox}.[13-14]. Furthermore, the C_μ of TiO_2 films provides information about the CB position. Thus, V_{oc} of PVs can be analyzed by C_μ as a function of V_F, where V_F is the corrected voltage that is the value of applied voltage excluding the voltage drop at total series resistance in the device.[15] Figure S14a shows the C_μ of PVs with TiO_2/QDs photoanodes as a function of V_F measured by IS. Interestingly, the results of C_μ showed the same tendency as V_{oc} in PVs. This can be explained by the higher CB energy level of T30 as compared to T20 and the decreased potential of CB in PT20 and PT30 due to the SPL. The energy level results of the TiO_2 films in Figure 4a strongly support the above argument. The recombination resistance (R_{rec}) and the transport resistance (R_t) were obtained from IS measurements, and recombination

was suppressed by the SPL on both PT20 and PT30. At the same time, the SPL reduced the transport resistance of TiO_2. R_t also trends with the electric conductivity of TiO_2 films (Figure S14c and Figure 5c).

Table S1. Photoelectrochemical Performance of Metal Oxide/QD Heterostructured Photoanodes for Hydrogen Generation.

Materials	Method	Sacrificial agent	Light intensity	Active area	PEC performance	References
TiO_2/CdS/CdSe	CBD	0.24M Na_2S, 0.35 M Na_2SO_3	1 sun	1 cm^2	14.9 mA/cm^2 (-0.6 V Ag/AgCl)	16
ZnO/CdS+ZnO/CdSe	CBD	0.25 M Na2S, 0.35M Na_2SO_3	1 sun	0.18 cm^2	12 mA/cm^2 (0.4 V Ag/AgCl)	17
TiO_2/PbS/Mn-CdS	SILAR	0.25 M Na_2S, 0.35 M Na_2SO_3	1 sun	0.25 cm^2	22.1 mA/cm^2 (0.6 V RHE)	18
TiO_2/CdSe	SILAR	0.24 M Na_2S, 0.35M Na_2SO_3	1 sun	0.25 cm^2	16.2 mA/cm^2 (0.5 V Ag/AgCl)	19
TiO_2/PbS/CdS	SILAR	0.5 M Na_2S, 0.5M Na_2SO_3	1 sun	-	6 mA/cm^2 (0.4 V RHE)	20
SnO_2/CdS	SILAR	0.25 M Na_2S, 0.35 M Na_2SO_3	1 sun	-	9.9 mA/cm^2 (0 V SCE)	21
TiO_2/PbS	SILAR	0.25 M Na_2S, 0.35 M Na_2SO_3	1 sun	0.16 cm^2	11.2 mA/cm^2 (0.2 V RHE)	22
TiO_2/CdS	SILAR	0.25 M Na_2S, 0.35M Na_2SO_3, 0.1M KCl	1 sun	-	2.31 mA/cm^2 (0 V Ag/AgCl)	23
TiO_2/SiO_2/CdS	SILAR	0.5 M KOH	1 sun	1.0 cm^2	4.6 mA/cm^2 (0 V Ag/AgCl)	24
TiO_2/CdSe/CdSSe/CdS	SILAR	0.25 M Na_2S, 0.35M Na_2SO_3	1 sun	0.1 - 0.4 cm^2	11.5 mA/cm^2 (0.6 V RHE)	25
TiO_2/CdS/CdSe	SILAR	0.25 M Na_2S, 0.35 M Na_2SO_3	1 sun	-	15.9 mA/cm^2 (1.0 V RHE)	26
TiO_2/PbS/CdS	SILAR	0.25 M Na_2S, 0.35 M Na_2SO_3	1 sun	1.33 cm^2	14.43 mA/cm^2 (0.8 V RHE)	**This work**

The photoelectrochemical performances of metal oxide/QD heterostructured photoanodes for hydrogen generation are listed in Table S1. Our results exhibited outstanding performance even with a relatively large active area (1.33 cm^2).

Table S2. Light-Harvesting Efficiency (η_{lh}) of TiO_2/PbS-CdS QDs Heterostructured Photoanodes Calibrated from IPCE at 480 nm.

**Samples**	**η_{lh} (%)**
T20	96.28
PT20	94.74
T30	95.08
PT30	93.36

REFERENCES

(1) Armstrong, A. R.; Armstrong, G.; Canales, J.; Bruce, P. G. TiO_2-B Nanowires. *Angew. Chem., Int. Ed.* **2004,** *43*, 2286-2288.

(2) Kobayashi, M.; Petrykin, V. V.; Kakihana, M.; Tomita, K.; Yoshimura, M. One-Step Synthesis of TiO_2(B) Nanoparticles from a Water-Soluble Titanium Complex. *Chem. Mater.* **2007,** *19*, 5373-5376.

(3) Chimupala, Y.; Junploy, P.; Hardcastle, T.; Westwood, A.; Scott, A.; Johnson, B.; Brydson, R. Universal Synthesis Method for Mixed Phase TiO_2(B)/Anatase TiO_2 Thin Films on Substrates via a Modified Low Pressure Chemical Vapour Deposition (LPCVD) Route. *J. Mater. Chem. A* **2016,** *4*, 5685-5699.

(4) Armstrong, A. R.; Armstrong, G.; Canales, J.; García, R.; Bruce, P. G. Lithium-Ion Intercalation into TiO_2-B Nanowires. *Adv. Mater.* **2005,** *17*, 862-865.

(5) Wang, G.; Wang, Q.; Lu, W.; Li, J. Photoelectrochemical Study on Charge Transfer Properties of TiO_2−B Nanowires with an Application as Humidity Sensors *J. Phys. Chem. B* **2006,** *110*, 22029-22034.

(6) van de Lagemaat, J.; Benkstein, K. D.; Frank, A. J. Relation between Particle Coordination Number and Porosity in Nanoparticle Films: Implications to Dye-Sensitized Solar Cells. *J. Phys. Chem. B* **2001,** *105*, 12433-12436.

(7) Hagfeldt, A.; Boschloo, G.; Sun, L.; Kloo, L.; Pettersson, H. Dye-Sensitized Solar Cells. *Chem. Rev.* **2010,** *110*, 6595-6663.

(8) Kweon, D. H.; Okyay, M. S.; Kim, S.-J.; Jeon, J.-P.; Noh, H.-J.; Park, N.; Mahmood, J.; Baek, J.-B. Ruthenium Anchored on Carbon Nanotube Electrocatalyst for Hydrogen Production with Enhanced Faradaic Efficiency. *Nat. Commun.* **2020,** *11*, 1278.

(9) Gimenez, S.; Dunn, H. K.; Rodenas, P.; Fabregat-Santiago, F.; Miralles, S. G.; Barea, E. M.; Trevisan, R.; Guerrero, A.; Bisquert, J. Carrier Density and Interfacial Kinetics of Mesoporous TiO_2 in Aqueous Electrolyte Determined by Impedance Spectroscopy. *J. Electroanal. Chem.* **2012,** *668*, 119-125.

(10) Bisquert, J.; Fabregat-Santiago, F.; Mora-Seró, I.; Garcia-Belmonte, G.; Barea, E. M.; Palomares, E. A Review of Recent Results on Electrochemical Determination of the Density of Electronic States of Nanostructured Metal-Oxide Semiconductors and Organic Hole Conductors. *Inorg. Chim. Acta* **2008,** *361*, 684-698.

(11) Park, K.; Zhang, Q.; Myers, D.; Cao, G. Charge Transport Properties in TiO_2 Network with Different Particle Sizes for Dye Sensitized Solar Cells. *ACS Appl. Mater. Interfaces* **2013,** *5*, 1044-1052.

(12) Fabregat-Santiago, F.; Garcia-Belmonte, G.; Mora-Seró, I.; Bisquert, J. Characterization of Nanostructured Hybrid and Organic Solar Cells by Impedance Spectroscopy. *Phys. Chem. Chem. Phys.* **2011,** *13*, 9083-9118.

(13) Kim, T.-Y.; Song, D.; Barea, E. M.; Lee, J. H.; Kim, Y. R.; Cho, W.; Lee, S.; Rahman, M. M.; Bisquert, J.; Kang, Y. S. Origin of High Open-Circuit Voltage in Solid State Dye-Sensitized Solar Cells Employing Polymer Electrolyte. *Nano Energy* **2016,** *28*, 455-461.

(14) Bai, Y.; Zhang, J.; Wang, Y.; Zhang, M.; Wang, P. Lithium-Modulated Conduction Band Edge Shifts and Charge-Transfer Dynamics in Dye-Sensitized Solar Cells Based on a Dicyanamide Ionic Liquid. *Langmuir* **2011,** *27*, 4749-4755.

(15) Raga, S. R.; Barea, E. M.; Fabregat-Santiago, F. Analysis of the Origin of Open Circuit Voltage in Dye Solar Cells. *J. Phys. Chem. Lett.* **2012,** *3*, 1629-1634.

(16) Lee, Y.-L.; Chi, C.-F.; Liau, S.-Y. CdS/CdSe Co-Sensitized TiO_2 Photoelectrode for Efficient Hydrogen Generation in a Photoelectrochemical Cell. *Chem. Mater.* **2010,** *22*, 922-

927.
(17) Wang, G.; Yang, X.; Qian, F.; Zhang, J. Z.; Li, Y. Double-Sided CdS and CdSe Quantum Dot Co-Sensitized ZnO Nanowire Arrays for Photoelectrochemical Hydrogen Generation. *Nano Lett.* **2010,** *10*, 1088-1092.
(18) Kim, J.-Y.; Jang, Y. J.; Park, J.; Kim, J.; Kang, J. S.; Chung, D. Y.; Sung, Y.-E.; Lee, C.; Lee, J. S.; Ko, M. J. Highly Loaded PbS/Mn-Doped CdS Quantum Dots for Dual Application in Solar-to-Electrical and Solar-to-Chemical Energy Conversion. *Appl. Catal. B* **2018,** *227*, 409-417.
(19) Kim, K.; Kim, M.-J.; Kim, S.-I.; Jang, J.-H. Towards Visible Light Hydrogen Generation: Quantum Dot-Sensitization via Efficient Light Harvesting of Hybrid-TiO_2. *Sci. Rep.* **2013,** *3*, 3330.
(20) Trevisan, R.; Rodenas, P.; Gonzalez-Pedro, V.; Sima, C.; Sanchez, R. S.; Barea, E. M.; Mora-Sero, I.; Fabregat-Santiago, F.; Gimenez, S. Harnessing Infrared Photons for Photoelectrochemical Hydrogen Generation. A PbS Quantum Dot Based "Quasi-Artificial Leaf". *J. Phys. Chem. Lett.* **2013,** *4*, 141-146.
(21) Zhang, Z.; Gao, C.; Wu, Z.; Han, W.; Wang, Y.; Fu, W.; Li, X.; Xie, E. Toward Efficient Photoelectrochemical Water-Splitting by Using Screw-Like SnO_2 Nanostructures as Photoanode after Being Decorated with CdS Quantum Dots. *Nano Energy* **2016,** *19*, 318-327.
(22) Jin, L.; AlOtaibi, B.; Benetti, D.; Li, S.; Zhao, H.; Mi, Z.; Vomiero, A.; Rosei, F. Near-Infrared Colloidal Quantum Dots for Efficient and Durable Photoelectrochemical Solar-Driven Hydrogen Production. *Adv. Sci.* **2016,** *3*, 1500345.
(23) Ding, D.; Zhou, B.; Liu, S.; Zhu, G.; Meng, X.; Yang, J.; Fu, W.; Yang, H. A Facile Approach for Photoelectrochemical Performance Enhancement of CdS QD-Sensitized TiO_2 via Decorating {001} Facet-Exposed Nano-Polyhedrons onto Nanotubes. *RSC Adv.* **2017,** *7*,

36902-36908.

(24) Li, L.; Chen, R.; Zhu, X.; Liao, Q.; Ye, D.; Zhang, B.; He, X.; Jiao, L.; Feng, H.; Zhang, W. A Ternary Hybrid CdS/SiO_2/TiO_2 Photoanode with Enhanced Photoelectrochemical Activity. *Renew. Energy* **2018,** *127*, 524-530.

(25) Liu, G.; Sun, B.; Li, H.; Wang, Y.; Zhao, H. Integration of Photoelectrochemical Devices and Luminescent Solar Concentrators Based on Giant Quantum Dots for Highly Stable Hydrogen Generation. *J. Mater. Chem. A* **2019,** *7*, 18529-18537.

(26) Selopal, G. S.; Mohammadnezhad, M.; Navarro-Pardo, F.; Vidal, F.; Zhao, H.; Wang, Z. M.; Rosei, F. A Colloidal Heterostructured Quantum Dot Sensitized Carbon Nanotube–TiO_2 Hybrid Photoanode for High Efficiency Hydrogen Generation. *Nanoscale Horiz.* **2019,** *4*, 404-414.
